# Foveal Density and Multi-Domain OCTA Biomarkers May Help Identify Preclinical Diabetic Microvasculopathy in Type 2 Diabetes Mellitus

**DOI:** 10.3390/medicina62061153

**Published:** 2026-06-13

**Authors:** Marko Zlatanović, Maja L. J. Živković, Nevena Zlatanović, Mladen Brzaković, Mihailo Jovanović

**Affiliations:** 1 Ophthalmology Clinic, University Clinical Center Niš, Bulevar Dr Zorana Đinđića 48, 18000 Nis, Serbia; drzlatanovicmarko@gmail.com; 2 Department of Ophthalmology, Faculty of Medicine, University of Niš, Bulevar Dr Zorana Đinđića 81, 18000 Nis, Serbia; 3 Community Health Center Niš in Niš, Vojvode Tankosića 15, 18000 Nis, Serbia; drnevenazlatanovic@gmail.com; 4 Special Hospital for Ophthalmology “Clinic Maja”, Vizantijski Bulevar 8, 18000 Nis, Serbia; brzi.92@hotmail.com; 5 Department of Ophthalmology, Faculty of Medicine, University of Kragujevac, Svetozara Markovića 69, 34000 Kragujevac, Serbia; drmihailojovanovic@gmail.com; 6 Ophthalmology Clinic, University Clinical Center Kragujevac, Zmaj Jovina 30, 34000 Kragujevac, Serbia

**Keywords:** diabetic retinopathy, OCT angiography, FD-300, foveal density, preclinical DR, deep capillary plexus, machine learning, SHAP, metabolic memory

## Abstract

*Background and Objectives*: Type 2 diabetes mellitus (T2DM) causes retinal microvascular changes that precede clinically apparent diabetic retinopathy (DR). We aimed to identify which optical coherence tomography angiography (OCTA) biomarkers best distinguish eyes with T2DM without clinical DR from healthy controls and to evaluate machine learning classifiers trained on a comprehensive 68-parameter OCTA panel. *Materials and Methods:* In this prospective case–control study, 80 patients with T2DM without clinical DR and 33 controls underwent 3 × 3 mm macular OCTA using an Optovue RTVue Avanti System. After outlier screening, 221 eyes (155 T2DM, 66 controls) were analyzed. Sixty-eight OCTA parameters were extracted, covering FAZ morphometry (including foveal density FD-300), SCP and DCP vessel density and layer thickness, outer-retina and choriocapillaris flow, and a full retinal-thickness map. Between-group comparisons used the Mann–Whitney U test with Benjamini–Hochberg FDR correction. Logistic regression, random forest, and XGBoost classifiers were evaluated with patient-grouped 10-fold cross-validation; feature importance was quantified via SHAP. *Results:* Forty-two of 68 parameters reached FDR significance (q < 0.05). Deep capillary plexus vessel density was the most discriminative family (whole image rb = −0.66, q = 2.5 × 10^−13^; parafovea rb = −0.64). FD-300 was reduced in T2DM (median 47.55% vs. 51.86%; rb = −0.57; q = 1.0 × 10^−10^) and emerged as the top SHAP feature (mean |SHAP| = 0.81). FAZ circularity decreased without FAZ-area enlargement, and outer-retina flow was paradoxically elevated (rb = +0.39), consistent with a projection artifact. XGBoost using all 68 features achieved a patient-grouped cross-validated AUC of approximately 0.91, compared with 0.85 for conventional SCP + DCP whole-image density. No parameter correlated with current HbA1c in T2DM (all q > 0.98), and the well-controlled (<7%) and poorly controlled (≥7%) subgroups were indistinguishable across five of six principal biomarkers, consistent with metabolic memory. FD-300 remained independent after adjustment for hypertension, hyperlipidemia, and age (OR = 0.76; 95% CI 0.69–0.84; *p* < 0.001). *Conclusions*: A multi-compartment OCTA panel outperforms conventional two-layer vessel-density metrics in detecting preclinical diabetic microvasculopathy, although external validation is required before clinical use. FD-300 is the single most informative biomarker, while choriocapillaris and retinal thickness measures provide complementary, compartment-specific signals. Because the OCTA signature is decoupled from the current HbA1c, screening should not be deferred in well-controlled T2DM.

## 1. Introduction

Diabetic retinopathy (DR) is the leading microvascular complication of diabetes mellitus and the principal cause of preventable blindness in working-age adults worldwide. The global number of adults living with diabetes rose to approximately 537 million in 2021 and is projected to reach 783 million by 2045, with most growth driven by type 2 diabetes mellitus (T2DM) [1]. A 2021 meta-analysis estimated the global prevalence of any DR among people with diabetes at 22.3%, with vision-threatening forms affecting 6.2% and corresponding to more than 100 million individuals with DR worldwide; the number with any DR is projected to exceed 160 million by 2045 [2]. Longitudinal data indicate that cumulative incidence rises steeply with disease duration—by 20 years after diagnosis, the majority of T2DM patients display some degree of retinopathy, and a substantial fraction progress to sight-threatening disease if untreated [3]. The direct and indirect societal burden, in terms of lost productive years, rehabilitation costs, and demand on ophthalmic services, remains considerable and is expected to intensify over the coming decades [4].

The pathogenesis of DR is now understood as a slowly evolving neurovascular disease rather than a purely microvascular one, integrating biochemical, hemodynamic, neuronal, and inflammatory components [5]. Chronic hyperglycemia drives flux through the polyol pathway, accelerates the formation of advanced glycation end-products, activates protein kinase C, and upregulates vascular endothelial growth factor (VEGF) and inflammatory mediators, including interleukin-6, tumor necrosis factor α, and intercellular adhesion molecule 1, collectively producing pericyte loss, endothelial dysfunction, basement-membrane thickening, and breakdown of the inner blood–retinal barrier [6,7]. Progressive capillary nonperfusion creates ischemic territories that further stimulate VEGF secretion and, eventually, pathological neovascularization. Clinically, DR is graded by the International Clinical Diabetic Retinopathy Severity Scale into non-proliferative (NPDR)–mild, moderate, and severe, based on microaneurysms, intraretinal hemorrhages, venous beading, and intraretinal microvascular abnormalities (IRMA)–and proliferative (PDR) stages, while diabetic macular edema (DME) can occur at any stage and is now stratified as center-involving or non-center-involving on optical coherence tomography (OCT) [8]. Importantly, inner-retinal neurodegeneration and subclinical microvascular dysfunction have been shown to precede detectable fundoscopic lesions, and updated disease-staging proposals have therefore begun to incorporate neurovascular and OCT-angiographic findings to capture pathology that the fundoscopic definition alone misses [9].

The most consistently replicated risk factors for DR are diabetes duration and chronic glycemic burden, typically indexed by glycated hemoglobin (HbA1c) [3]. Long-term follow-up of the DCCT/EDIC cohort has shown that each 1% reduction in HbA1c is associated with approximately a 35% reduction in microvascular complications and that the protective effect of tight glycemic control persists for years beyond the period of active intervention—a phenomenon termed metabolic memory [10]. Arterial hypertension and dyslipidemia independently accelerate DR progression, and blood-pressure control reduces retinopathy incidence in T2DM cohorts [11]. Additional modulating factors include pregnancy, chronic kidney disease, anemia, and obstructive sleep apnea; genome-wide association studies have further identified susceptibility loci that contribute modestly to individual risk, with genes involved in inflammation, endothelial signaling, and pericyte biology featuring prominently [12]. Despite this extensive knowledge of modifiable risk factors, even well-controlled patients accumulate microvascular injury over time, underscoring the clinical importance of detection strategies capable of identifying the earliest structural changes before functional deficits emerge.

Contemporary DR screening relies predominantly on color fundus photography, supplemented by ultra-widefield imaging and spectral-domain OCT to detect DME. Autonomous artificial-intelligence triage systems—EyeArt, IDx-DR, and related platforms—have demonstrated sensitivity exceeding 90% for referable DR and are now deployed in multiple national screening programs [13,14]. These tools, however, are inherently constrained by fundus image resolution and by the definition of DR they are trained on, namely, the presence of visible microvascular lesions. Optical coherence tomography angiography (OCTA) has transformed retinal vascular assessment over the past decade by providing depth-resolved, dye-free, motion-contrast visualization of the superficial capillary plexus (SCP), the deep capillary plexus (DCP), the outer retina, and the choriocapillaris at micron-level resolution [15,16]. Commercial platforms now report, directly from built-in analytic software, quantitative vessel density (VD) in user-selected regions, foveal avascular zone (FAZ) morphometry, layer thickness, and foveal density (FD-300)—the vessel density within a 300 µm annulus immediately surrounding the FAZ, a compartment that targets the parafoveal capillary ring most vulnerable to early ischemic injury [17,18].

Applied to T2DM cohorts without clinical DR, OCTA has repeatedly demonstrated that preclinical microvasculopathy is prevalent rather than marginal: enlargement and shape distortion of the FAZ, parafoveal vessel-density reductions in both SCP and DCP, and early alterations in outer-retinal and choriocapillaris perfusion have each been described in eyes with entirely normal fundoscopy [19,20,21]. The DCP has emerged as a particularly vulnerable compartment because of its end-arteriolar perfusion pattern and its dual dependence on inner-retinal oxygenation and vertical anastomotic flow from the SCP. Yet three important gaps persist in the current literature. First, most published analyses focus on a handful of preselected biomarkers, so the relative discriminative weight of each compartment within a single, coherent panel remains unclear. Second, multiple-testing correction across the full quantitative OCTA output is inconsistently applied, inflating false-positive rates in exploratory reports. Third, although machine learning models have begun to appear in OCTA-based DR classification, very few studies combine a comprehensive multi-compartment feature set with modern explanation techniques such as SHAP values, which are necessary to translate classifier output into clinically interpretable biomarker rankings. The present study was designed to address these three gaps directly, through a comprehensive 68-parameter OCTA analysis with FDR-corrected statistics, cross-validated supervised classifiers, and SHAP-based feature ranking, in a prospective T2DM cohort without clinical DR and age-matched healthy controls.

## 2. Materials and Methods

### 2.1. Study Design and Participants

This prospective, observational, case–control study was conducted at the University Eye Clinic, Clinical Centre Niš, Serbia, from March to October 2019. The study was conducted and reported in accordance with the STROBE statement for observational studies [22]. The protocol was approved by the Institutional Ethics Committee of the Clinical Centre Niš (Decision No. 12-526-2/5 of 24 January 2019) and adhered to the Declaration of Helsinki. Written informed consent was obtained from all participants prior to enrollment. Consecutive patients referred from the University Diabetes Clinic with biochemically confirmed T2DM (per the American Diabetes Association 2019 diagnostic criteria) and without clinically apparent diabetic retinopathy on dilated fundus examination were invited to participate. An age- and sex-matched control group of healthy volunteers was recruited in parallel from hospital staff and accompanying relatives.

Eligible participants were aged 40–70 years and, for the diabetic cohort, had a known T2DM duration of 1–20 years. All participants provided informed consent and had sufficiently clear ocular media for high-quality OCTA acquisition. Exclusion criteria included any ophthalmoscopic sign of diabetic retinopathy or diabetic macular edema on spectral-domain OCT; spherical-equivalent refractive error exceeding 6 diopters; glaucoma or ocular hypertension; prior intravitreal pharmacotherapy or panretinal laser treatment; age-related macular degeneration; epiretinal membrane or vitreomacular traction; previous intraocular surgery other than uncomplicated cataract extraction; uncontrolled systemic hypertension (>160/100 mmHg at enrollment); and any neurological, hematological, or systemic inflammatory condition known to affect retinal microcirculation. Both eyes of each eligible participant were imaged and analyzed, yielding a planned cohort of 160 T2DM eyes from 80 patients and 66 control eyes from 33 volunteers.

A formal a priori sample-size calculation, based on an anticipated medium-to-large effect size for principal OCTA biomarkers (Cohen d ≈ 0.6), with two-sided α = 0.05 and power of 0.90 under a 2:1 allocation, indicated that at least 114 T2DM eyes and 57 control eyes would be required. The enrolled cohort therefore provides substantial power for the primary between-group comparisons and sufficient power for planned subgroup analyses.

### 2.2. Ophthalmic and Systemic Examination

Each participant underwent a standardized ophthalmic assessment, including best-corrected Snellen visual acuity (converted to logMAR), Goldmann applanation tonometry, slit-lamp biomicroscopy of the anterior segment, and dilated fundus examination with a 90-diopter non-contact lens after pupil dilation with 1% tropicamide. Spectral-domain OCT (Optovue RTVue Avanti) was performed in each eye to confirm the absence of diabetic macular edema and other macular pathology; eyes with intraretinal fluid, cystoid spaces, or a central subfield thickness greater than 300 µm were excluded from the analytic cohort. Systemic data collected by study personnel included age, sex, body mass index, systolic and diastolic blood pressure measured after a 10 min seated rest, glycated hemoglobin (HbA1c) expressed as a percentage on the NGSP scale, and self-reported history of arterial hypertension (HTA) and hyperlipidemia (HLP), including current treatment. For the T2DM group, diabetes duration and glucose-lowering therapy (oral hypoglycemic agents, insulin, or combination) were also recorded.

### 2.3. OCTA Imaging Protocol

Macular OCTA was performed on an Optovue RTVue Avanti System with AngioVue™ software (version 2016.1.0.26, Optovue Inc., Fremont, CA, USA) using the split-spectrum amplitude-decorrelation angiography (SSADA) algorithm. A 3 × 3 mm en-face scan centered on the fovea was acquired after pupil dilation, following the manufacturer’s standard protocol. All scans were obtained by a single experienced operator (M.Z.) to minimize inter-rater variability. Automated layer segmentation by the AngioVue software was verified slice-by-slice for each scan; segmentation errors confined to peripheral regions were manually corrected where feasible. Scans with uncorrectable segmentation failure, signal strength below 40 arbitrary units, visible motion or banding artifacts, projection artifacts extending into the outer-retinal slab, or centration offset exceeding 0.3 mm from the foveal center were rejected. Quality control and segmentation review were carried out by a masked senior grader (D.V.) who was unaware of group assignment.

### 2.4. OCTA Parameter Extraction

Sixty-eight quantitative parameters covering the full macular OCTA output were automatically extracted from the AngioVue AngioAnalytic module, with only four derived quantities computed post hoc, as described below. The parameter set was designed to represent all clinically relevant compartments of the retinal and choroidal microcirculation, rather than a preselected subset of traditionally reported indices.

Foveal avascular zone morphometry was characterized by the following four parameters: FAZ area (mm^2^), FAZ perimeter (mm), and two derived shape indices—the circularity index, defined as 4πA/P^2^ (with a value of 1 denoting a perfect circle) and its complement, the acircularity index, defined as 1 − 4πA/P^2^. The single perifoveal density metric, FD-300 (%), corresponds to the vessel density within the 300 µm annulus immediately surrounding the FAZ border; it is computed by AngioAnalytic as the ratio of signal-positive pixels to total pixels in this annulus, multiplied by 100, providing a direct quantification of parafoveal capillary filling adjacent to the foveal border.

The superficial capillary plexus was characterized by eleven vessel-density and eleven layer-thickness parameters measured across the same set of regional subdivisions: the whole 3 × 3 mm image, the superior and inferior hemispheres, the central foveal disc, the full parafoveal annulus, its superior and inferior hemispheres, and its four radial sectors (temporal, superior, nasal, and inferior). The deep capillary plexus was characterized by an identical set of eleven vessel-density parameters and an identical set of eleven layer-thickness parameters, yielding forty-four inner-retinal vascular parameters in total. Vessel density values were expressed as the percentage of signal-positive pixels within each region, and layer thickness values were reported in micrometers. Both were provided directly by the AngioAnalytic software without user intervention.

Outer-retinal perfusion was summarized by the flow area (mm^2^) within the avascular outer-retinal slab and by the flow ratio, computed as the flow area divided by the slab’s nominal select area. Analogous flow-area and flow-ratio parameters were extracted for the choriocapillaris slab, yielding four flow-based parameters that span the normally avascular and choroidal compartments. Finally, fifteen retinal-thickness measurements were drawn from the full Early Treatment Diabetic Retinopathy Study (ETDRS) grid—the central foveal subfield, the parafoveal annulus with its two hemispheres and four radial sectors, and the perifoveal annulus with the same regional subdivision—providing a complete retinal-thickness map in micrometers.

All post-processing of the raw AngioAnalytic export was limited to the four derived quantities already noted (FAZ circularity, FAZ acircularity, outer-retina flow ratio, and choriocapillaris flow ratio). No additional binarization, skeletonization, or custom image processing was performed, ensuring that the parameter values reported here can be reproduced from the commercial software output alone.

### 2.5. Statistical Analysis

All statistical analyses were conducted in Python 3.11 using pandas, NumPy, SciPy, statsmodels, scikit-learn 1.3, XGBoost 2.0, and SHAP 0.44. Continuous variables were summarized with the median and interquartile range [Q1, Q3], because most OCTA parameters were non-normally distributed. Categorical variables were summarized with absolute and relative frequencies.

Because both eyes of each participant were imaged, the primary analyses treated each eye as the unit of analysis and accounted for the resulting within-subject correlation. Sensitivity analyses restricted to one randomly selected eye per participant were conducted for the principal biomarkers and yielded results comparable in magnitude and direction; therefore, the two-eye analysis is presented as the primary result, consistent with prior OCTA literature in T2DM cohorts.

Outlier screening was performed separately for each OCTA parameter using a 3 × IQR rule, with values exceeding Q3 + 3 × IQR or falling below Q1 − 3×IQR flagged as outliers. Eyes with five or more flagged parameters were excluded from the primary analysis, yielding an analytic cohort of 221 eyes (155 T2DM, 66 Control). Sensitivity analyses using more restrictive thresholds (≥3 or ≥4 flagged parameters) and no outlier removal yielded essentially unchanged patient-grouped cross-validated classifier AUC (range 0.90–0.91; Supplementary Table S7), confirming robustness to this choice.

Between-group comparisons of each OCTA parameter used the Mann–Whitney U test, and the rank-biserial correlation coefficient rb (range [−1, +1]; negative values indicating T2DM < Control) served as the effect-size metric. *p*-values across the 68 OCTA parameters were corrected for multiple testing using the Benjamini–Hochberg false-discovery-rate (FDR) procedure, and significance was declared at q < 0.05. Baseline demographic comparisons used the Mann–Whitney U test for continuous variables and Pearson’s chi-squared test for categorical variables, without FDR correction, given the small, pre-specified set of such comparisons.

Spearman rank correlations among the principal biomarkers, systemic variables (age, HbA1c), and the full OCTA panel were computed within the T2DM cohort and then FDR-corrected for each correlation set. A multivariable logistic regression was fitted with FD-300, HTA, HLP, and age as predictors of T2DM status to assess the independence of the OCTA signal from systemic confounders.

To evaluate the discriminative value of the full 68-parameter OCTA panel and compare it with clinically interpretable reduced panels, three supervised classifiers were trained on the full feature set, as follows: (a) L2-regularized logistic regression implemented in scikit-learn with standardization (StandardScaler) and inverse regularization C = 1.0; (b) random forest with 300 trees and default hyperparameters; and (c) gradient-boosted trees using XGBoost with 200 estimators, maximum depth 4, and learning rate 0.1. Classifier performance was estimated using patient-grouped 10-fold cross-validation (Section 2.6), and the area under the receiver operating characteristic (ROC) curve (AUC) was used as the primary metric. Two reference panels were also evaluated under the same cross-validation scheme: a conventional two-feature panel comprising the SCP and DCP whole-image vessel densities, and an integrative five-feature panel comprising FD-300, DCP parafoveal vessel density, FAZ circularity, choriocapillaris flow area, and outer-retina flow area. Feature importance for the XGBoost model was quantified using SHAP (SHapley Additive exPlanations) values [23], and features were ranked by their mean absolute SHAP contribution. Dependence plots were generated for the top-ranked features to visualize their individual effects on the classifier output.

To assess whether current glycemic control further stratifies the OCTA phenotype, the T2DM cohort was divided into well-controlled (T2DM-WC) and poorly controlled (T2DM-PC) subgroups based on the American Diabetes Association HbA1c < 7% threshold. Principal biomarkers were then compared across Control, T2DM-WC, and T2DM-PC using pairwise Mann–Whitney U tests with FDR correction applied to each parameter.

### 2.6. Unit of Analysis, Inter-Eye Dependence, and Statistical Limitations

Because both eyes of each participant were imaged, the dataset has a two-level structure in which the two eyes of a given participant are not statistically independent. The primary analyses were conducted at the eye level, which preserves the full information of the cohort and is consistent with the prevailing OCTA literature in T2DM. To ensure that this choice did not bias the principal results, inter-eye dependence was addressed by three complementary procedures.

First, all machine learning classifiers were evaluated using patient-grouped cross-validation (StratifiedGroupKFold, 10 folds, fixed random seed 42), in which both eyes of any participant are always assigned to the same fold. This prevents the two correlated eyes of a participant from being split between the training and test sets, which would otherwise produce optimistically biased performance estimates. Patient-grouped cross-validation is treated as the primary scheme throughout; conventional stratified cross-validation is also reported for comparison.

Second, the multivariable logistic regression for T2DM status (Section 3.14) was refitted using cluster-robust (Huber–White sandwich) standard errors, with the participant as the clustering unit. This leaves the coefficient estimates unchanged but adjusts the standard errors and confidence intervals to account for the within-participant correlation between the two eyes.

Third, a one-eye-per-participant sensitivity analysis was performed, in which the multivariable model was refitted on a single, randomly selected eye from each participant; this was repeated over 50 independent random selections.

Generalized estimating equations (GEE) and mixed-effects models with a participant-level random intercept were considered but are not applicable to the present outcome. The classification and regression outcome—T2DM versus control status—is identical for both eyes of a participant and therefore constant within each cluster. Consequently, there is no within-participant outcome variance to estimate for an exchangeable working correlation or a random intercept, and such models are not identifiable for this outcome (in our data, the GEE exchangeable working-correlation parameter degenerated to approximately 1). Cluster-robust inference and one-eye-per-participant analysis are the appropriate tools for handling inter-eye dependence when the outcome is cluster-constant, and patient-grouped cross-validation is the corresponding safeguard for the supervised classifiers.

## 3. Results

### 3.1. Cohort Characteristics and Baseline Comparisons

**Figure 1:**
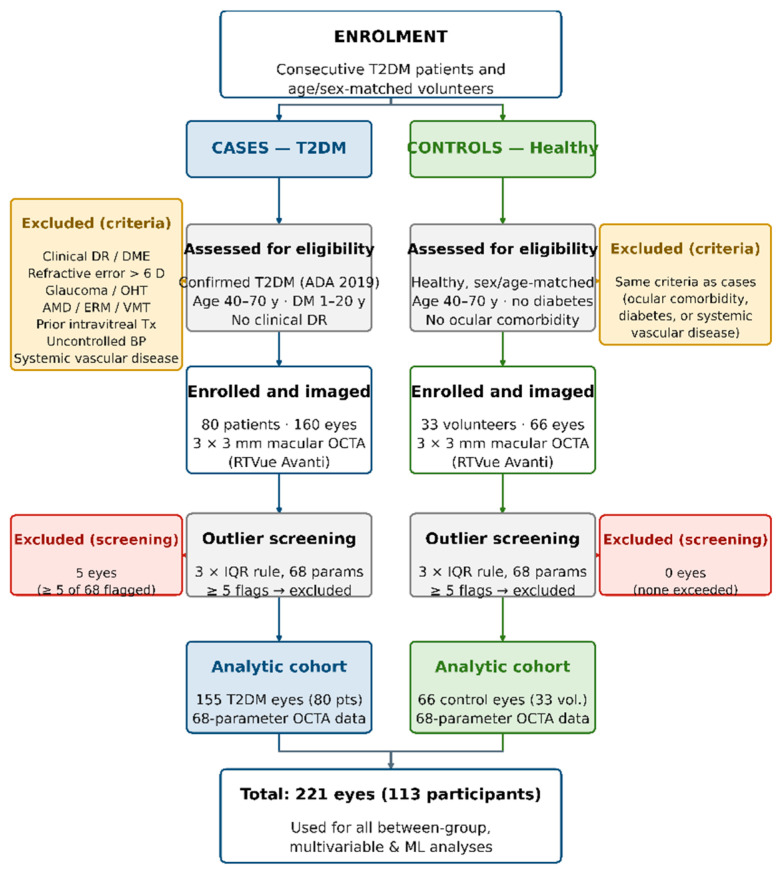
Participant flow through enrollment, OCTA imaging, and outlier screening.

Of the 226 eyes originally imaged (160 T2DM eyes from 80 patients and 66 control eyes from 33 volunteers), 5 eyes were excluded by the outlier-screening procedure described in Section 2.5, resulting in a primary analytic cohort of 221 eyes (155 T2DM and 66 control). Full demographic and clinical characteristics are presented in Table 1. The flow of participants through enrollment, imaging, and outlier screening is summarized in Figure 1.

Despite age-matching at recruitment, the T2DM group was slightly older than the control group (mean 58.7 ± 13.0 years vs. 55.3 ± 12.3 years; *p* = 0.031), with a comparable sex distribution (M/F 80/75 in T2DM vs. 30/36 in Control; *p* = 0.490). HbA1c differed markedly between the two groups (7.96 ± 2.36% vs. 5.05 ± 0.56%; *p* = 1.15 × 10^−28^), confirming the distinction between diabetic and non-diabetic status. The range of HbA1c in the T2DM group (5.2–16.7%) spanned patients classified as well-controlled to poorly controlled, providing a wide metabolic gradient for testing OCTA biomarkers. Arterial hypertension was substantially more frequent in T2DM (54.8% vs. 24.2%; *p* = 5.55 × 10^−5^), and hyperlipidemia was also more common (18.1% vs. 6.1%; *p* = 0.035), reflecting the expected clustering of cardiometabolic comorbidities in the diabetic population.

### 3.2. Global Pattern of Octa Alterations Across 68 Parameters

**Figure 2:**
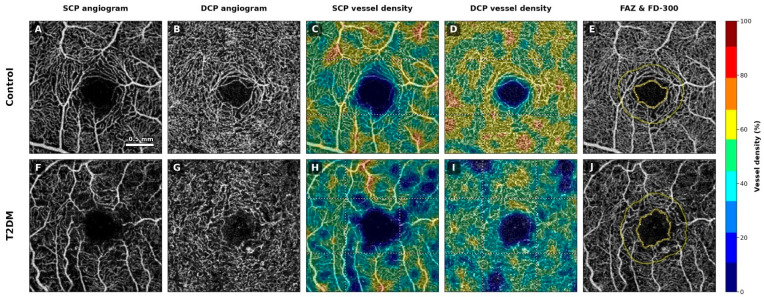
Representative 3 × 3 mm macular OCTA scans (Optovue RTVue Avanti, AngioVue; central ⟨2.43 × 2.43⟩ mm shown) of a control eye (Row A) and a T2DM patient without clinical retinopathy (Row B): (**A**,**F**) superficial capillary plexus (SCP) angiogram; (**B**,**G**) deep capillary plexus (DCP) angiogram; (**C**,**H**) SCP vessel-density map; (**D**,**I**) DCP vessel-density map; (**E**,**J**) SCP angiogram overlaid with the foveal avascular zone (FAZ, inner contour) and the 300 µm annulus used to compute FD-300 (outer ring). Control eye (74 y, male, SSI ⟨x⟩/10): SCP VD 50.8%, DCP VD 56.0%, FAZ area 0.190 mm^2^, FAZ circularity 0.766, FD-300 54.11%. T2DM eye (63 y, female, SSI ⟨x⟩/10; HbA1c 5.7%, diabetes duration ⟨y⟩ y): SCP VD 42.0%, DCP VD 43.1%, FAZ area 0.285 mm^2^, FAZ circularity 0.725, FD-300 46.5%. Relative to the control, the T2DM eye shows reduced deep-plexus vessel density, a less circular FAZ contour, and lower FD-300. Color bar = vessel density (%); scale bar (panel A) = 0.5 mm, applies to all panels.

Representative enface OCTA images from a control eye and a T2DM eye without clinical retinopathy are shown in Figure 2 (control, Row A; T2DM, Row B). In the control eye (74-year-old male; signal strength index [SSI] ⟨x⟩/10), the superficial and deep capillary plexuses appeared dense and uniform (Figure 2A,B), the color-coded density maps were dominated by mid-to-high values (Figure 2C,D), and the foveal avascular zone showed a smooth, regular contour (Figure 2E). In the T2DM eye (63-year-old female; HbA1c 5.7%; diabetes duration ⟨y⟩ years; SSI ⟨x⟩/10), the deep capillary plexus showed visibly sparser perifoveal capillaries (Figure 2G) and more extensive low-density regions on the corresponding map (Figure 2I). Whole-image deep vessel density was markedly reduced relative to the control (43.1% vs. 56.0%); the superficial plexus was also reduced but to a lesser degree (Figure 2F,H; 42.0% vs. 50.8%). The foveal avascular zone showed a visibly more irregular, less circular contour (Figure 2J; circularity 0.725 vs. 0.766), and FD-300 was lower (46.5% vs. 54.11%). At the cohort level, FAZ area did not differ significantly between groups, but FAZ circularity was consistently reduced in T2DM (Section 3.4); the contour change is the FAZ pattern best illustrated by these representative cases. Together, these eyes illustrate the predominant pattern observed across the cohort (Table 2), in which deep-plexus vessel density and the perifoveal microvascular measures (FD-300 and FAZ circularity) were the parameters most consistently altered in the T2DM group.

Each of the 68 OCTA parameters was compared between the T2DM and Control groups using Mann–Whitney U tests, with Benjamini–Hochberg FDR correction applied across the full 68-parameter panel. Forty-two parameters (61.8%) reached the q < 0.05 significance threshold. The distribution of significant findings and effect-size magnitudes across biomarker families was strongly non-uniform, as summarized below and visualized in the volcano plot (Figure S1). The plot displays rank-biserial effect size on the horizontal axis against −log_10_(q) on the vertical axis for each parameter, color-coded by family.

Significant findings were concentrated in the vascular families. In the deep capillary plexus, 10 of 11 vessel-density parameters were significant, with the largest effect sizes in the panel (rank-biserial rb −0.58 to −0.66). The superficial capillary plexus showed the same regional pattern at a smaller magnitude (10 of 11 vessel-density parameters significant; rb −0.46 to −0.57), as did FD-300 (rb −0.57). Both outer-retina flow parameters were significant, with positive effect sizes (rb +0.39)—the only positive-rb findings in the inner-retinal panel—and were isolated on the right-hand side of the volcano plot. Six of eleven parameters in each of the SCP thickness and DCP thickness families were significant, with smaller magnitudes (|rb| up to 0.27). Three of fifteen retinal-thickness map subfields reached significance, with the central foveal subfield showing a positive effect size (+0.26) in contrast to the two parafoveal or perifoveal subfields, which were negative. Two of four FAZ parameters reached significance (circularity and acircularity; |rb| = 0.24), while the classical FAZ area and perimeter metrics did not. Both choriocapillaris parameters were significant, with |rb| values of approximately 0.22–0.25. The full 68-parameter results table with medians, interquartile ranges, rank-biserial effect sizes, *p*-values, and FDR-adjusted q-values are provided in Supplementary Table S1, and the top 15 FDR-significant parameters ranked by |rb| are listed in Supplementary Table S2. Supplementary Figure S2 complements this view by ranking every parameter by its univariate AUC for T2DM-versus-Control discrimination, again confirming DCP vessel density dominance at the top of the ranking.

### 3.3. Foveal Density (FD-300)

**Figure 3:**
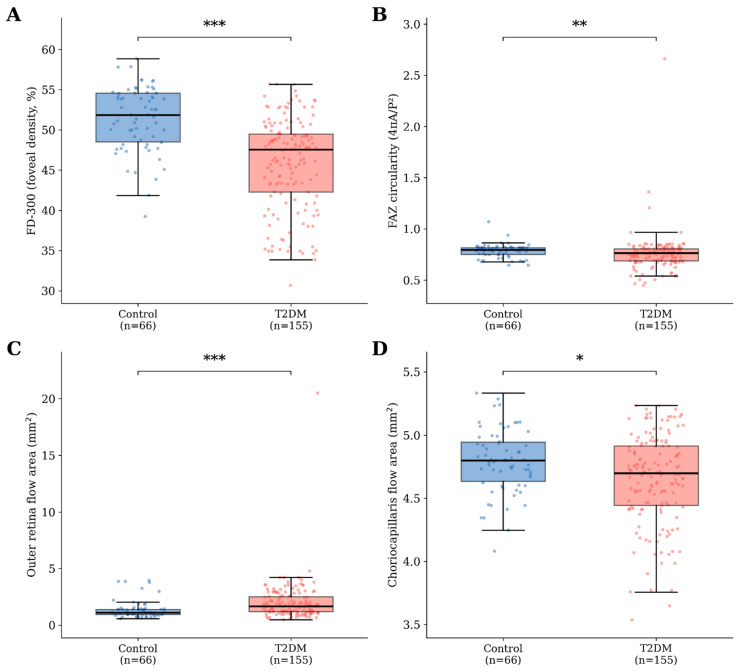
Distribution of four principal OCTA biomarkers in T2DM versus Control eyes. Four-panel boxplot showing (**A**) FD-300 foveal density, (**B**) FAZ circularity index, (**C**) outer-retina flow area, and (**D**) choriocapillaris flow area. In each panel, the boxes represent the interquartile range (Q1–Q3), the horizontal line inside each box is the median, and the whiskers extend to 1.5× IQR beyond Q1/Q3. Individual eye values are overlaid as jittered dots. Sample sizes are n = 66 for the Control group and n = 155 for the T2DM group. Statistical significance of the between-group difference (Mann–Whitney U) is shown above each panel using the conventional notation: * q < 0.05; ** q < 0.01; *** q < 0.001 after Benjamini–Hochberg FDR correction across the 68-parameter OCTA panel.

FD-300 was substantially lower in T2DM eyes, with a median of 47.55% [Q1–Q3: 42.30–49.50] compared with 51.86% [48.52–54.56] in control eyes (rb = −0.57, *p* = 1.78 × 10^−11^, q = 1.01 × 10^−10^). This reduction represents a median drop of 4.31 percentage points, equivalent to approximately 8% of the control median. The distribution of individual values is shown in Figure 3A, where non-overlapping interquartile boxes and tightly clustered whiskers confirm the large separation between groups. Within the T2DM group, FD-300 values fell as low as 30%, whereas no control eye fell below 39.5%, indicating that the most severely affected T2DM eyes occupy a parafoveal vessel-density range not seen in any control eye. As reported in the full panel summary (Supplementary Table S1) and in Table 2, FD-300 was the second most discriminative single OCTA parameter when ranked by effect size, tied with SCP parafoveal vessel density and exceeded only by members of the DCP VD family.

### 3.4. Foveal Avascular Zone Morphometry

The classical FAZ area and perimeter metrics showed only modest, non-significant trends toward smaller values in T2DM (FAZ area median 0.271 mm^2^ vs. 0.304 mm^2^, rb = −0.17, q = 0.070; FAZ perimeter 2.151 mm vs. 2.237 mm, rb = −0.10, q = 0.277). In contrast, the two shape-based FAZ indices reached significance in opposite directions: FAZ circularity was reduced (0.764 vs. 0.795; rb = −0.24, q = 0.010) and its complement, FAZ acircularity, was correspondingly elevated (0.236 vs. 0.205; rb = +0.24, q = 0.010). The distribution of circularity values across groups is shown in Figure 3B, where the T2DM group shows broader dispersion and a downward shift in the median, with several eyes exhibiting circularity values below 0.6 that are not observed in controls. This dissociation between size and shape—with FAZ geometry distorted without overall enlargement—is discussed in the Section 4. Exact medians, effect sizes, and q-values for all four FAZ parameters are provided in Table 2.

### 3.5. Vessel Density in the Superficial and Deep Capillary Plexuses

Vessel density was reduced in T2DM across both capillary plexuses, with substantially larger effect sizes in the DCP. In the DCP, whole-image vessel density was 48.10% [45.10–51.75] in T2DM versus 54.45% [52.00–55.80] in controls (rb = −0.66, q = 2.50 × 10^−13^), and the corresponding parafoveal measurement was 50.20% [46.85–54.05] versus 56.30% [54.40–57.98] (rb = −0.64, q = 7.57 × 10^−13^). Every parafoveal sector of the DCP—temporal, superior, nasal, and inferior—as well as both the superior and inferior parafoveal hemispheres, was significantly reduced in T2DM at q < 10^−10^ (sector effect sizes ranging from −0.58 to −0.64). The central foveal subfield of the DCP, which normally exhibits physiological avascularity around the FAZ, did not differ between groups (rb = −0.05, q = 0.576). The same regional pattern held in the SCP at a smaller magnitude: the whole-image SCP vessel density was 43.30% [39.05–46.80] in T2DM versus 47.60% [45.70–49.00] in controls (rb = −0.52, q = 3.62 × 10^−9^); parafoveal SCP VD was 45.70% [42.10–49.95] versus 51.10% [49.10–52.55] (rb = −0.57, q = 1.01 × 10^−10^); and every parafoveal sector reached FDR significance at q < 10^−7^ (rb −0.46 to −0.55). The SCP foveal subfield was again not significantly different (rb = +0.05, q = 0.549). All twenty-two individual SCP and DCP VD results are tabulated in Supplementary Table S1.

**Figure 4:**
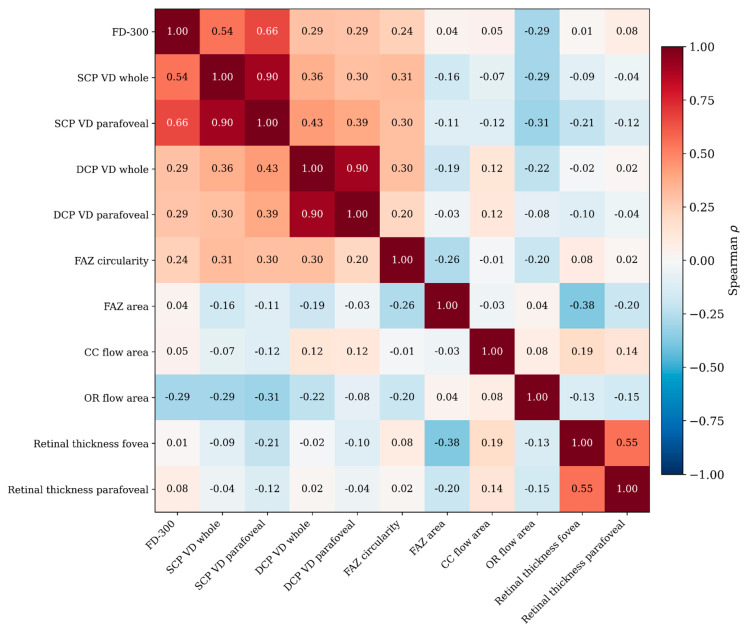
Spearman correlation heatmap of the 11 principal OCTA biomarkers in the T2DM cohort. The 11 × 11 matrix shows pairwise Spearman ρ coefficients for the biomarkers listed on the axes, computed for the T2DM subgroup (*n* = 155). The color scale ranges from blue (ρ = −1) through white (ρ = 0) to red (ρ = +1). Numerical ρ values are shown in each cell.

The within-cohort relationship between FD-300 and parafoveal SCP vessel density is shown in Supplementary Figure S3 as a scatter plot. Both diagnostic groups show strong positive Spearman correlations (ρ = +0.56 in controls, ρ = +0.67 in T2DM), but the T2DM cloud is shifted toward lower values on both axes. The broader pattern of covariance within the T2DM cohort is summarized in the 11 × 11 correlation heatmap in Figure 4, where the three inner-retinal vascular families (FD-300, SCP VD variants, DCP VD variants) cluster together, with ρ values ranging from +0.43 to +0.93. The FAZ, outer-retina, choriocapillaris, and retinal-thickness families form separate blocks with weaker or inverse cross-family correlations.

### 3.6. Layer Thickness and Retinal Thickness Map

Layer thickness in both capillary plexuses was significantly reduced in T2DM, but to a lesser extent than the reductions in vessel density. Five of the eleven SCP thickness parameters reached FDR significance (SCP thickness parafovea superior, inferior, S-hemi, I-hemi, and the parafoveal whole; rb −0.19 to −0.24, q= 0.012–0.038), and six of the eleven DCP thickness parameters reached significance (DCP thickness parafovea as a whole had the largest effect at rb = −0.27, q = 0.005). In contrast, the central foveal thickness in both plexuses (SCP foveal thickness rb = +0.26, q = 0.006; DCP foveal thickness rb = +0.20, q = 0.032) was increased in T2DM—an opposite-direction effect from the parafoveal reductions.

This pattern was mirrored in the ETDRS retinal-thickness map. Only three of the fifteen retinal-thickness subfields reached significance: retinal thickness in the foveal subfield was paradoxically increased in T2DM (median 265 µm vs. 253 µm; rb = +0.26, q = 0.005), whereas retinal thickness in the parafoveal superior sector (313 µm vs. 323.5 µm; rb = −0.28, q = 0.003) and in the perifoveal superior sector (287 µm vs. 293 µm; rb = −0.23, q = 0.018) was reduced. The remaining twelve ETDRS subfields did not reach significance after FDR correction. Together with the thickness findings in the SCP and DCP layers, these observations suggest a topographically heterogeneous T2DM effect on retinal thickness, as follows: a mild fovea-centered thickening coexisting with parafoveal and perifoveal thinning, consistent with early subclinical fluid redistribution superimposed on inner-retinal neurodegeneration. Full retinal-thickness and layer-thickness results are provided in Supplementary Table S1.

### 3.7. Outer-Retina and Choriocapillaris Flow

Both outer-retina flow parameters showed a counterintuitive, significant elevation in T2DM. The outer-retina flow area was 1.68 mm^2^ [1.21–2.51] in T2DM versus 1.11 mm^2^ [0.95–1.40] in controls (rb = +0.39, q = 1.34 × 10^−5^), and the outer-retina flow ratio was 0.239 [0.172–0.357] versus 0.157 [0.135–0.199] (rb = +0.39, q = 1.30 × 10^−5^). This elevation of OCTA-detectable flow signal in a normally avascular compartment is the only major positive-rb result in the inner-retinal vascular panel (Supplementary Figure S1) and is shown in Figure 3C, where the T2DM distribution is right-shifted with a long upper tail.

In contrast, the choriocapillaris showed modest but significant reductions in flow in T2DM. Choriocapillaris flow area was 4.70 mm^2^ [4.44–4.92] in T2DM versus 4.80 mm^2^ [4.63–4.95] in controls (rb = −0.22, q = 0.021), and the choriocapillaris flow ratio was 0.661 [0.625–0.693] versus 0.678 [0.656–0.699] (rb = −0.25, q = 0.009). The distribution of choriocapillaris flow area values is shown in Figure 3D. Although the median difference is small relative to the spread, the separation is statistically significant after multiple-testing correction and aligns with the growing literature reporting early choroidal involvement in T2DM.

### 3.8. Ranking of the Principal Biomarkers by Effect Size

To convey the hierarchy of biomarker magnitudes in a single view, Supplementary Table S2 ranks the top fifteen FDR-significant OCTA parameters by absolute rank-biserial effect size. The top ten positions are occupied exclusively by DCP vessel-density parameters (|rb| 0.58–0.66), followed by SCP parafoveal VD and FD-300, tied at rb ≈ −0.57, then by SCP parafoveal inferior VD, SCP whole-image VD, and SCP parafoveal S-hemi VD. This ranking is consistent with the univariate AUC ranking shown in Supplementary Figure S2 and Table S3—where eight of the top ten AUCs belong to the DCP VD family and FD-300 occupies the twelfth slot with AUC = 0.786—confirming that the relative discriminative contribution of each biomarker is stable across statistical testing frameworks.

The full hierarchy of pairwise inter-parameter associations within the T2DM cohort, summarized in Supplementary Table S4 (top 30 |ρ| pairs), confirms and quantitatively refines the three-domain structure. Three tiers of correlation strength are evident. First, within-family correlations dominate (|ρ| > 0.85), reflecting the expected geometric coherence of sectoral measurements within a single en-face slab: SCP VD whole-image versus parafoveal (ρ = +0.93), DCP VD whole-image versus parafoveal (ρ = +0.92), DCP superior versus inferior hemispheric VD (ρ = +0.88), and analogous within-family couplings for FAZ area–perimeter (ρ = +0.95), CC flow area–ratio (ρ = +0.95), and OR flow area–ratio (ρ = +0.97). Second, cross-family vascular correlations occupy an intermediate range (|ρ| 0.42–0.71): the strongest such cross-family signal is FD-300 versus SCP parafoveal VD (ρ = +0.71; q = 1.3 × 10^−23^), positioning FD-300 as an integrative summary of parafoveal capillary filling rather than an independent biomarker. Third, parafoveal SCP and DCP slab thicknesses correlate with full-thickness retinal thickness (|ρ| 0.68–0.74), a coherent structural relationship. Separately, outer-retina flow area shows weak inverse correlations with SCP parafoveal VD (ρ = −0.33), DCP parafoveal VD (ρ = −0.27), and FD-300 (ρ = −0.27), consistent with the compensatory-redistribution interpretation of outer-retinal hyperperfusion already evident in Figure 1.

### 3.9. Correlations with HbA1c and Age Within the T2DM Cohort

Within the T2DM cohort, none of the 68 OCTA parameters showed a significant correlation with HbA1c measured at enrollment. Despite a wide HbA1c range (5.2–16.7%; mean 7.96 ± 2.36%), all 68 Spearman correlations were weak (raw |ρ| ≤ 0.14), and none survived FDR correction (all q ≥ 0.97; full correlation table in Supplementary Table S4, top 25 in Supplementary Table S5). The strongest individual correlations–none reaching significance after correction for multiple testing–were observed for retinal thickness at the parafovea (ρ = −0.14, *p* = 0.097), outer-retina flow area (ρ = +0.13, *p* = 0.120), and outer-retina flow ratio (ρ = +0.12, *p* = 0.131). FD-300, which showed the largest between-group effect, had a within-cohort correlation with HbA1c of only ρ = +0.05 (*p* = 0.524).

Age showed weak, expected-direction correlations with SCP-based and FD-300 measures in the T2DM cohort (older age associated with lower perifoveal vessel density), with the strongest age effect in FD-300 (ρ = −0.22; *p* = 0.012 within T2DM). In all cases, the magnitude of the age effect was substantially smaller than the diabetes effect reported in Section 3.3, Section 3.4, Section 3.5, Section 3.6 and Section 3.7 and did not account for the observed between-group differences.

### 3.10. Hypertension Stratification Within the T2DM Cohort

Within the T2DM group, 85 eyes had concurrent arterial hypertension (T2DM + HTA) and 70 did not (T2DM–HTA). A head-to-head comparison of these two subgroups, with FDR correction applied across the panel of principal biomarkers, identified a small but coherent subset of parameters showing additional HTA-associated compromise (Supplementary Table S6). Choriocapillaris flow area and choriocapillaris flow ratio were both significantly lower in the T2DM + HTA subgroup than in the T2DM–HTA subgroup (CC flow area *p* = 0.011, q = 0.034; CC flow ratio *p* = 0.006, q = 0.034), and FAZ area was significantly larger in T2DM + HTA (*p* = 0.008, q = 0.034). Retinal thickness at the parafovea was also modestly different (*p* = 0.002, q = 0.025), being slightly higher in the T2DM–HTA subgroup. In contrast, the dominant diabetes-driven biomarkers—FD-300, SCP and DCP vessel density, and outer-retina flow—did not differ between the two T2DM subgroups (all q > 0.40), indicating that these signals are attributable to diabetes itself rather than to accompanying hypertension. The pattern of selective HTA-associated choroidal and FAZ-geometric compromise is consistent with the known susceptibility of the choriocapillaris to combined diabetic–hypertensive stress.

### 3.11. Metabolic Memory: Control Versus Well-Controlled Versus Poorly-Controlled T2DM

**Figure 5:**
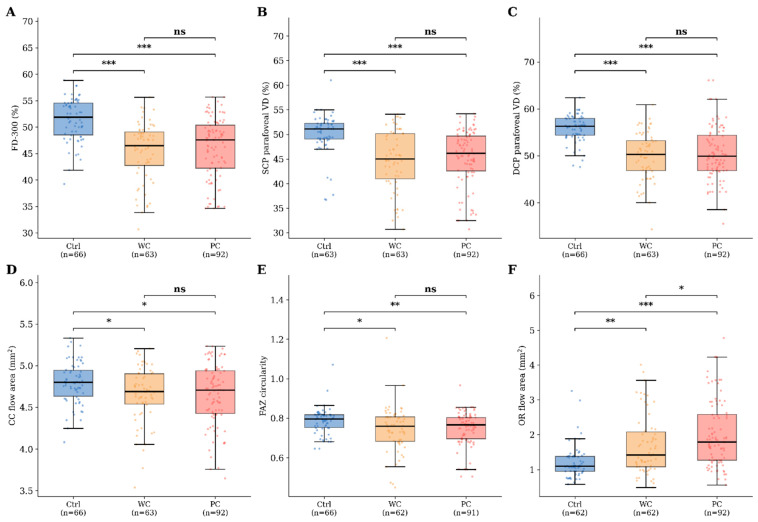
Metabolic-memory analysis: Control versus T2DM well-controlled (HbA1c < 7%) versus T2DM poorly-controlled (HbA1c ≥ 7%) for six principal biomarkers. Six-panel boxplot showing (**A**) FD-300, (**B**) SCP parafoveal vessel density, (**C**) DCP parafoveal vessel density, (**D**) choriocapillaris flow area, (**E**) FAZ circularity, and (**F**) outer-retina flow area. Group sample sizes are Control n = 66, T2DM-WC n = 63, T2DM-PC n = 92. Boxes and whiskers follow the same conventions as Figure 1, and individual eye values are overlaid as jittered dots. Pairwise significance brackets are shown above each box for Control–WC, Control–PC, and WC–PC comparisons (Mann–Whitney U), using the standard annotation (* *p* < 0.05; ** *p* < 0.01; *** *p* < 0.001; ns = not significant).

Using the American Diabetes Association HbA1c threshold of 7%, the T2DM cohort was stratified into well-controlled (T2DM-WC; HbA1c < 7%, n = 63) and poorly controlled (T2DM-PC; HbA1c ≥ 7%, n = 92) subgroups. Six principal biomarkers were compared across the three groups (Control, T2DM-WC, T2DM-PC) using pairwise Mann–Whitney U tests, with significance annotations on each panel. The results are shown in Figure 5.

For every inner-retinal vascular biomarker, both T2DM subgroups were strongly separated from the control group (Control vs. T2DM-WC and Control vs. T2DM-PC were highly significant at *p* < 10^−7^ for FD-300, SCP parafoveal VD, and DCP parafoveal VD), but the two T2DM subgroups were statistically indistinguishable from one another (T2DM-WC vs. T2DM-PC: FD-300 *p* = 0.45, SCP parafoveal VD *p* = 0.61, DCP parafoveal VD *p* = 0.87). The choriocapillaris flow area and FAZ circularity followed the same pattern, with highly significant Control-versus-T2DM differences in both subgroups and non-significant WC-versus-PC differences (CC flow area pWC–PC = 0.96; FAZ circularity pWC–PC = 0.77). The outer-retina flow area was the only biomarker that showed a modest additional elevation in the T2DM-PC subgroup compared with T2DM-WC (*p* = 0.048); the direction of this effect—higher flow in poorer glycemic control—is compatible with an enhanced projection-artifact signal in a more severely affected inner-retinal circulation. Taken together, five of the six biomarkers showed no detectable differences between the well-controlled and poorly controlled subgroups, even though the two subgroups differed in HbA1c by 2–10 percentage points. This pattern is consistent with metabolic memory: once structural OCTA abnormalities are established, they do not appear to be rapidly reversed by improvements in current glycemic control.

### 3.12. Machine-Learning Classification Performance

**Figure 6:**
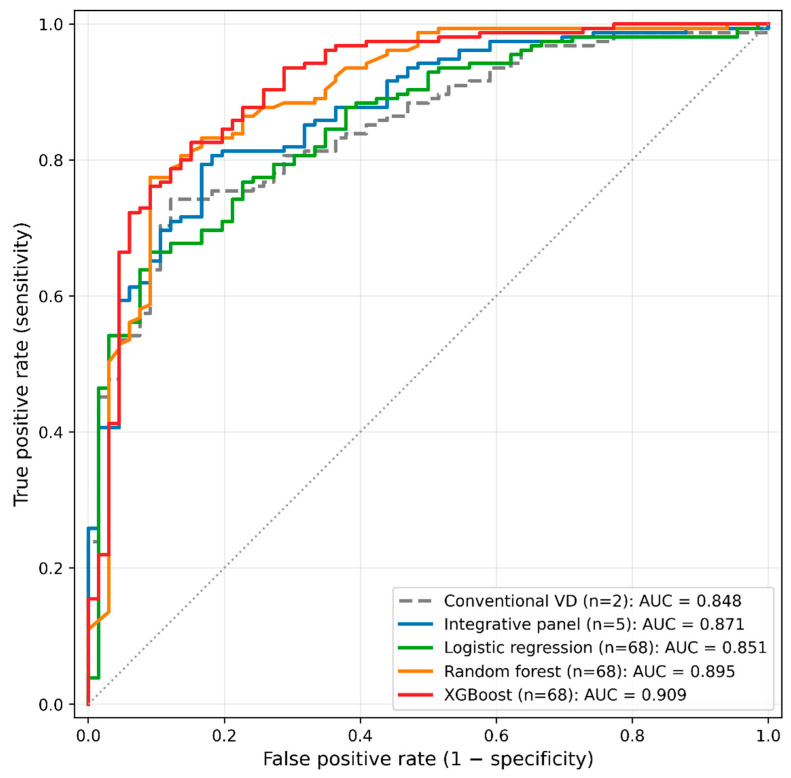
Receiver-operating-characteristic (ROC) curves for five classifiers on the T2DM-versus-Control task under patient-grouped cross-validation. All curves are derived from the pooled out-of-fold predictions of patient-grouped 10-fold cross-validation (StratifiedGroupKFold; both eyes of a participant assigned to the same fold) on the 221-eye analytic cohort, using the full 68-feature OCTA panel for the three supervised classifiers (logistic regression, random forest, XGBoost) and the corresponding reduced feature subsets for the two reference panels (conventional two-feature SCP + DCP whole-image vessel density; integrative five-feature panel comprising FD-300, DCP parafoveal vessel density, FAZ circularity, choriocapillaris flow area, and outer-retina flow area). The pooled out-of-fold AUC is shown in the legend for each model. The diagonal dotted line represents a non-informative classifier (AUC = 0.5).

The discriminative value of the full 68-parameter OCTA panel was evaluated using patient-grouped 10-fold cross-validation (Section 2.6) across three supervised classifier families, with conventional stratified cross-validation reported for comparison. Full numerical results for both schemes are presented in Table 3, and the patient-grouped receiver operating characteristic curves are shown in Figure 6.

Under patient-grouped cross-validation, the primary XGBoost model trained on all 68 OCTA features achieved a cross-validated AUC of approximately 0.91 (pooled out-of-fold AUC 0.909; mean AUC 0.88–0.93 across five random fold assignments). The random forest performed comparably (AUC 0.909), whereas the logistic regression model on the same 68 features achieved a lower AUC of 0.864, again indicating that linear aggregation of many collinear features adds little discriminative power. Among the reduced reference panels, the integrative five-feature panel reached an AUC of 0.877, and the conventional two-feature SCP + DCP whole-image vessel-density panel reached 0.865, while FD-300 alone yielded 0.792.

The non-linear ensemble classifiers retained a discriminative advantage of approximately 0.06 AUC over the conventional two-feature baseline under patient-grouped cross-validation; this gap was somewhat smaller than under conventional cross-validation and was consistent across five random fold assignments, though the between-fold standard deviation under patient grouping (~0.07–0.09) remained appreciable. The patient-grouped estimates were close to the conventional stratified cross-validation values (XGBoost pooled out-of-fold AUC 0.909 vs. 0.926; Table 3), indicating that the inter-eye information leakage targeted by patient grouping had only a minor inflationary effect in this cohort; patient-grouped cross-validation is, nevertheless, treated as the primary scheme because it provides the methodologically defensible estimate of generalization to new participants.

At the Youden-optimal classification threshold derived from the patient-grouped out-of-fold predictions, the XGBoost model achieved a sensitivity of 82.6%, specificity of 84.8%, positive predictive value of 92.8%, and negative predictive value of 67.5%. A sensitivity analysis across outlier-screening thresholds confirmed that the patient-grouped XGBoost AUC was robust to this choice, remaining in the range 0.90–0.91 (Supplementary Table S7). The relative ordering of the eight pre-specified logistic-regression panels (A–H) under patient-grouped cross-validation is shown in Supplementary Figure S4 and tabulated in Supplementary Table S8, confirming that the integrative panel H remains the strongest member of that family.

### 3.13. SHAP-Based Feature Ranking

**Figure 7:**
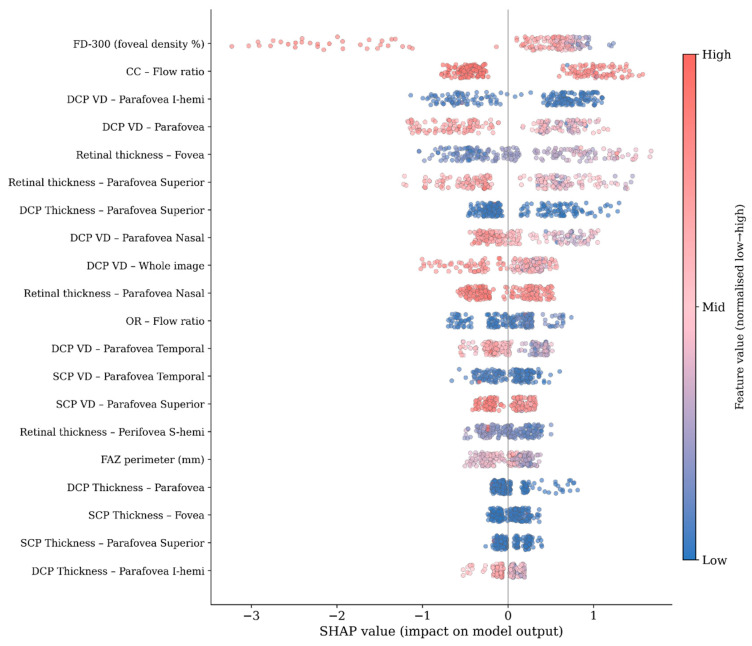
SHAP summary plot for the XGBoost classifier, top 20 features. Each row corresponds to a single OCTA feature, ranked from top (highest mean |SHAP|) to bottom. For each row, each dot represents one eye from the analytic cohort of 221 eyes (155 T2DM and 66 control eyes); the horizontal position gives the SHAP value for that feature on that eye (negative → classifier output shifted toward Control; positive → shifted toward T2DM), and the dot color encodes the normalized raw feature value (blue = low, pink/red = high).

To translate the XGBoost classifier output into a clinically interpretable biomarker ranking, SHAP values were computed for each feature in each eye in the cohort, and features were ranked by their mean absolute SHAP values. The full top-20 ranking, including per-sample SHAP scatter, is shown in Figure 7; the corresponding numerical values are tabulated in Supplementary Table S9.

FD-300 emerged as the most influential feature in the XGBoost model (mean |SHAP| = 0.813), despite not being the highest-ranked feature based on univariate AUC. This result indicates that the model leverages FD-300 in a non-linear, threshold-sensitive manner–assigning large negative SHAP contributions (toward the T2DM class) to eyes below a critical FD-300 level and large positive contributions (toward Control) to eyes above it–in a way that simpler linear aggregation cannot capture. Choriocapillaris flow ratio ranked second (0.722), followed by DCP parafoveal I-hemi vessel density (0.659), DCP parafoveal vessel density (0.651), retinal thickness at the fovea (0.580), and retinal thickness at the parafoveal superior sector (0.579). The remaining top-20 features spanned all major biomarker families (DCP thickness, SCP VD sectors, outer-retinal flow ratio, FAZ perimeter, and additional retinal-thickness sectors), indicating that each OCTA compartment contributes to the classifier’s decision.

Per-feature dependence plots for the top six features, showing individual-eye SHAP values against the underlying parameter values, with diagnosis-colored scatter points, are presented in Supplementary Figure S5. Each dependence curve shows a broadly monotonic relationship with a pronounced transition around the diagnostic decision boundary–FD-300 near 50%, choriocapillaris flow ratio near 0.67, and DCP parafoveal VD near 54%–consistent with the presence of biologically meaningful thresholds separating preclinical-DR from control eyes rather than a continuous dose–response relationship.

### 3.14. Multivariable Logistic Regression for the Independence of the FD-300 Signal

To assess whether the FD-300 signal persists after adjustment for systemic confounders, a multivariable logistic regression was fitted with T2DM status as the binary outcome and FD-300, arterial hypertension, hyperlipidemia, and age as predictors (Table 4). FD-300 remained an independent predictor of T2DM status (β = −0.273 per percentage-point, *p* < 0.001; odds ratio 0.761, 95% CI 0.691–0.838), indicating that each one-percentage-point reduction in FD-300 is associated with approximately a 31% increase in the odds of being classified as T2DM after controlling for HTA, HLP, and age. Arterial hypertension was also an independent predictor (OR = 4.26, 95% CI 1.90–9.54; *p* < 0.001), as expected given its strong association with diabetic microvasculopathy. In contrast, HLP (OR = 2.23, *p* = 0.229) and age (OR = 0.995 per year, *p* = 0.702) were not independent predictors after adjustment.

## 4. Discussion

### 4.1. Principal Findings

This prospective case–control study systematically characterized 68 quantitative OCTA parameters in eyes with T2DM without clinical retinopathy and identified four coherent biomarker patterns. Forty-two of the sixty-eight parameters reached FDR significance, with the deep capillary plexus vessel-density family dominating the effect-size ranking (|rb| = 0.58–0.66), followed by the superficial plexus, foveal density (FD-300), choriocapillaris flow, outer-retina flow, FAZ shape indices, and retinal-thickness map subfields. A supervised XGBoost model trained on all 68 parameters achieved a cross-validated AUC of 0.927 ± 0.049, substantially above the two-feature conventional vessel-density baseline (0.848 ± 0.076). FD-300 emerged as the single most influential feature in the SHAP ranking. None of the 68 OCTA parameters was FDR—significantly correlated with current HbA1c within the T2DM cohort, and well-controlled and poorly-controlled T2DM subgroups were statistically indistinguishable on five of six principal biomarkers, a pattern consistent with metabolic memory. Multivariable logistic regression confirmed that FD-300 remained an independent predictor of T2DM status after adjustment for hypertension, hyperlipidemia, and age.

### 4.2. The Deep Capillary Plexus Is the Most Vulnerable Compartment

The most striking finding of the present study is the disproportionate impact of preclinical diabetic microvasculopathy on the deep capillary plexus. Every parafoveal DCP VD parameter reached q < 10^−10^, with effect sizes |rb| = 0.58–0.66, and the top ten positions in the effect-size ranking were occupied exclusively by DCP VD variants. This pattern has been reported across a growing number of OCTA cohorts. Nesper et al. demonstrated that DCP-derived geometric perfusion deficits discriminated clinically referable DR with an AUC of 0.965, significantly exceeding that of SCP-based metrics [24]. Hsieh et al., in a large multi-severity cohort, reported that artifact-free quantitative DCP metrics were more strongly associated with visual function than SCP metrics, and that projection-artifact correction was essential for accurate DCP quantification [25]. A recent systematic review of OCTA biomarkers in preclinical DR concluded that DCP changes are the most consistently reported across studies, with decreased vessel density and increased non-perfusion area detected prior to the onset of clinical retinopathy [20].

The anatomical basis for DCP vulnerability is well established. The DCP lies at the watershed between the retinal and choroidal circulations, receives end-arteriolar perfusion via vertical anastomoses from the SCP, and supplies the photoreceptor inner segments and the outer plexiform layer through a high-resistance, low-flow network that is poorly tolerant of microcirculatory impairment. Our data reinforce this interpretation: DCP VD reductions are uniformly larger than SCP VD reductions in the same eyes (parafoveal DCP VD rb = −0.64 vs. parafoveal SCP VD rb = −0.57), and the ten-fold gradient in q-value between the two plexuses (qDCP = 7.57 × 10^−13^ versus qSCP = 1.01 × 10^−10^ for the parafoveal measurement) implies that DCP capillary dropout has progressed further than SCP dropout at a stage before any fundoscopic abnormality. Our finding that the foveal subfield of the DCP is statistically indistinguishable among groups (rb = −0.05, q = 0.576) is also biologically consistent: this subfield corresponds to the normally avascular foveal pit, so vessel-density differences cannot arise there regardless of disease severity. The same argument explains the null result at the SCP foveal subfield.

### 4.3. FD-300 as the Leading Perifoveal Biomarker

FD-300 measures vessel density within the 300 µm annulus immediately surrounding the FAZ and, therefore, captures the integrity of the parafoveal terminal capillary ring, which is anatomically most exposed to ischemic injury [18,26]. In our cohort, FD-300 was reduced by a median of 4.3 percentage points (8% of the control median; rb = −0.57), tying with parafoveal SCP VD as the second most discriminative perifoveal biomarker after DCP VD. Notably, FD-300 emerged as the most influential feature in the XGBoost SHAP ranking, despite not being the highest-ranked biomarker based on univariate AUC. This dissociation indicates that the XGBoost model leverages FD-300 in a nonlinear, threshold-sensitive manner rather than through linear aggregation; the SHAP dependence plot shows a pronounced transition near FD-300 ≈ 50%, consistent with a biologically meaningful cutoff separating preclinical-DR from control eyes.

The landmark study by Rosen et al. [18] established that perifoveal perfused capillary density distinguishes T2DM without DR from controls, with effect sizes comparable to those observed here. Subsequent work has replicated this finding in both spectral-domain and swept-source OCTA [27,28]. The recent descriptive review by Strauss et al. [20] highlighted peri-FAZ vessel density (with FD-300 as the commercial AngioAnalytic implementation) as one of the earliest detectable markers of preclinical DR, noting that it often precedes overt FAZ enlargement. Our finding of a substantial FD-300 reduction in the absence of significant FAZ area change is fully consistent with this chronology: the annular perifoveal ring loses capillaries through diffuse dropout before the FAZ itself becomes measurably enlarged. From a clinical standpoint, FD-300 has the practical advantage of being generated automatically by AngioAnalytic without user intervention or post-processing, making it more readily transferable to routine practice than research metrics that require custom image analysis.

A practical caveat concerns the cross-platform comparability of FD-300. FD-300 is computed using device-specific algorithms—here, the AngioAnalytic software on an SSADA-based spectral-domain platform—and absolute values are not directly interchangeable between spectral-domain and swept-source systems or across vendors because of differences in segmentation, decorrelation algorithms, and normative ranges. The direction and relative magnitude of the FD-300 reduction reported here are consistent with the peri-FAZ vessel-density literature across platforms, but platform-specific reference values would be required before any threshold-based clinical use.

### 4.4. FAZ Geometry: Shape Change Without Enlargement

Our cohort showed a clear dissociation between FAZ size and FAZ shape: neither FAZ area nor FAZ perimeter reached FDR significance, yet FAZ circularity was reduced (rb = −0.24, q = 0.010), and its complement, FAZ acircularity, was correspondingly elevated. This dissociation is consistent with a diffuse capillary dropout pattern that distorts the FAZ perimeter without substantially enlarging its total area. As capillary segments are lost at scattered locations around the FAZ border, the perimeter grows faster than the area, and the circularity index 4πA/P^2^ therefore decreases even when A is approximately preserved [10,29].

This pattern has been reported across multiple OCTA cohorts of T2DM without DR. Strauss and Gawęcki [20] noted that decreased perifoveal vessel density in T2DM without DR reflects diffuse parafoveal capillary dropout rather than FAZ enlargement. A systematic review of FAZ-based OCTA biomarkers in DR concluded that shape indices (circularity, acircularity, and axis ratio) are more sensitive than FAZ area alone for detecting early diabetic microvasculopathy, and that FAZ area differences often emerge only with progression to NPDR or PDR [29,30]. One practical advantage of shape indices is that they are unitless and, unlike FAZ area, do not require axial-length correction to account for individual retinal magnification [29]. Our data are therefore fully aligned with the current literature: circularity and acircularity detect the subtle diffuse capillary dropout that characterizes preclinical DR, whereas the classical area metric is too insensitive at this stage of the disease.

### 4.5. Outer-Retina Flow Reflects a Projection Artifact

One of the more counterintuitive findings of our analysis is the significantly elevated outer-retinal flow signal in T2DM eyes (rb = +0.39 for both flow area and flow ratio; q = 10^−5^). Because the outer-retinal slab is normally avascular, any flow signal detected within it in T2DM eyes is unlikely to represent true outer-retinal vessels: genuine outer-retinal neovascularization occurs only in proliferative DR, which was an exclusion criterion. The more plausible interpretation is a projection artifact, in which the motion-contrast signal from the overlying superficial capillary plexus is projected onto the outer-retinal slab.

Projection artifacts are among the most prevalent in OCTA imaging, affecting nearly 100% of OCTA images in some reports [31,32]. They are known to be amplified when the overlying vasculature is remodeled or dilated—as occurs in diabetes. Ashraf et al. quantified this effect directly and showed that eyes with moderate-to-severe NPDR had systematically higher pre-projection-artifact-removal vessel density in deeper slabs than eyes with less severe disease. This gradient disappeared after projection-artifact-removal (PAR) software was applied [31]. Because the AngioAnalytic software used in the present study applies only partial projection-artifact compensation, outer-retinal flow values in diabetic eyes should be interpreted primarily as an indirect marker of overlying inner-retinal vascular disturbance rather than as evidence of true outer-retinal vessels. The fact that outer-retinal flow was also the only biomarker showing a modest further elevation in T2DM-PC versus T2DM-WC is consistent with this interpretation: more severely affected inner-retinal circulation produces a more pronounced projected signal. Accordingly, we treat outer-retinal flow in the XGBoost and SHAP analyses as a useful but mechanistically indirect feature, and we recommend that future studies that replicate our findings apply full-volume PAR to resolve this interpretative ambiguity. More broadly, automated layer segmentation in spectral-domain OCTA is imperfect, particularly at the DCP–outer-retina boundary, and residual segmentation error compounds the projection-artifact signal; both factors reinforce the need for swept-source platforms with projection-resolved segmentation in confirmatory studies.

### 4.6. Choriocapillaris Involvement and the Hypertension Interaction

Choriocapillaris flow was modestly but significantly reduced in T2DM (CC flow area rb = −0.22, q = 0.021; CC flow ratio rb = −0.25, q = 0.009), and the choriocapillaris flow ratio ranked second in XGBoost SHAP feature importance. The HTA stratification analysis revealed a striking interaction: although the dominant inner-retinal biomarkers (FD-300, SCP/DCP VD, OR flow) did not differ between T2DM + HTA and T2DM–HTA subgroups (all q > 0.40), CC flow area and CC flow ratio were further reduced in T2DM + HTA (q = 0.034 for both), along with a larger FAZ area and a thinner parafoveal retina. This pattern indicates that choroidal perfusion is selectively susceptible to combined diabetic–hypertensive stress, beyond the isolated effect of diabetes.

Our findings align with growing evidence that choriocapillaris involvement is an integral rather than a peripheral component of diabetic eye disease. Wang et al. followed 1222 patients with T2DM for one year and demonstrated that each 1% increase in baseline CC flow deficit increased the odds of developing referable DR by 2.69-fold [33]. The 3-year longitudinal cohort of Chen et al. (n = 903) extended these findings, showing that CC flow deficit adds significant predictive value for DR progression and DME development beyond known systemic risk factors [34]. Vidal-Oliver et al. further reported that in T2DM without DR, CC flow deficit was the only OCTA parameter independently associated with disease duration after adjustment for age, sex, HbA1c, and hypertension [35]. Together with these data, our HTA-stratification results argue that choroidal involvement should be included as a dedicated compartment in any comprehensive OCTA biomarker panel for preclinical DR, and that incorporating CC metrics into screening models is likely to enhance detection of high-risk diabetic eyes with comorbid hypertension.

### 4.7. Retinal-Thickness Topography

The retinal-thickness map results were directionally heterogeneous. The central foveal subfield was paradoxically thicker in T2DM (median 265 µm vs. 253 µm; rb = +0.26, q = 0.005), whereas the parafoveal superior sector (313 vs. 323.5 µm; rb = −0.28, q = 0.003) and the perifoveal superior sector (287 vs. 293 µm; rb = −0.23, q = 0.018) were thinner in T2DM. A parallel pattern was observed in the SCP and DCP layer thicknesses: five of eleven SCP and six of eleven DCP parafoveal subfields reached FDR significance with negative rb, whereas the foveal subfield showed a positive rb in both layers.

Parafoveal and perifoveal thinning is most parsimoniously explained by early diabetic retinal neurodegeneration. Multiple OCT studies have now demonstrated thinning of the ganglion cell layer, retinal nerve fiber layer, and inner plexiform layer in T2DM without DR, with thinning progressing before or in parallel with vascular changes [5,36,37]. In recent evidence review for the Mary Tyler Moore Vision Initiative staging system, Channa et al. summarized this literature and concluded that inner-retinal thinning should be incorporated as a neurodegeneration biomarker in the next generation of DR staging [9]. The longitudinal OCTA study by Lynch et al. documented a progressive mGCIPL loss of 0.45 µm per year in eyes with early-stage DR, accompanied by corresponding OCTA microvascular impairment [38]. Our parafoveal thinning findings are fully compatible with this emerging view of preclinical DR as a combined neurodegenerative and microvascular process.

The paradoxical foveal thickening requires a different explanation. At the preclinical stage, overt diabetic macular edema is excluded by definition; yet subclinical fluid redistribution and very mild breakdown of the inner blood–retinal barrier have been documented on high-resolution OCT in diabetic eyes without funduscopically visible DR [39]. A recent analysis by De Clerck et al. reported that subclinical fluid accumulation of approximately 5–15 µm is detectable in the central foveal subfield of T2DM eyes years before DR becomes clinically apparent and may precede both the microaneurysm stage and the onset of frank DME [40]. In our cohort, the magnitude of foveal thickening (~12 µm) is consistent with this subclinical fluid redistribution mechanism, while the simultaneous thinning of the parafoveal/perifoveal ring reflects the inner retinal neurodegeneration described above. Together, these changes capture the two poles of early diabetic retinal disease in a single topographical map. Notably, foveal thickening was less pronounced in T2DM + HTA than in T2DM–HTA eyes, a finding consistent with hypertension-driven choroidal and retinal thinning partially offsetting subclinical fluid accumulation.

### 4.8. Decoupling from HbA1c and Metabolic Memory

A central and, in our view, clinically actionable finding is that the OCTA signature is almost entirely decoupled from current HbA1c in the T2DM cohort. None of the 68 parameters reached FDR significance against HbA1c; all within-cohort |ρ| values were ≤ 0.14, and FD-300 had a within-cohort HbA1c correlation of only ρ = +0.05 (*p* = 0.524). More strikingly, when the T2DM cohort was split at the ADA HbA1c threshold of 7%, the well-controlled and poorly controlled subgroups were statistically indistinguishable across five of six principal biomarkers, despite a between-subgroup HbA1c gradient spanning 2–10 percentage points. Control eyes, in contrast, were strongly separated from both T2DM subgroups at *p* < 10^−7^ for every inner-retinal vascular biomarker.

This pattern aligns with the metabolic-memory framework. The concept of metabolic memory, originally formalized by Lachin, Nathan, and colleagues in the DCCT/EDIC cohort, describes a long-term effect of historical glycemic burden on the risk of diabetic complications, independent of the individual’s current glycemic state [10,41]. Once cumulative hyperglycemic injury crosses a threshold that triggers oxidative stress, AGE deposition, epigenetic remodeling, and mitochondrial dysfunction in the retinal microcirculation, the resulting structural changes do not reverse quickly, even if glycemic control is subsequently improved [42,43]. Our cross-sectional OCTA data capture the structural imprint of accumulated injury, reflecting many years of HbA1c exposure rather than the single-timepoint HbA1c measured at enrollment. The practical implication is clear: achieving a current HbA1c < 7% in a patient with longstanding T2DM does not imply intact retinal microcirculation, and OCTA screening for preclinical DR should not be deferred in well-controlled patients. This stands in instructive contrast to functional measures such as real-time glycemic variability from continuous glucose monitoring, which have been reported to correlate with SCP vessel density in recent short-duration T1DM cohorts [44] but in our longer-duration T2DM cohort are decoupled from structural OCTA markers.

### 4.9. Machine-Learning Performance and Interpretability

The XGBoost model trained on the full 68-parameter OCTA panel achieved a patient-grouped cross-validated AUC of approximately 0.91 (pooled out-of-fold AUC 0.909), corresponding to sensitivity 82.6%, specificity 84.8%, PPV 92.8%, and NPV 67.5% at the Youden-optimal threshold, values appropriate for a screening-grade classifier. A sensitivity analysis across outlier-exclusion thresholds confirmed a stable AUC of 0.90–0.91. The primary gain over the conventional two-feature baseline (AUC 0.848) came from two sources, as follows: the inclusion of multi-compartment features that capture choroidal and structural (thickness) dimensions missed by a pure VD panel and the use of a non-linear tree-based learner capable of exploiting the inter-block interactions evident in the correlation heatmap. The logistic regression model on the same 68 features achieved an AUC of only 0.856, essentially equal to the two-feature baseline, confirming that linear aggregation of many collinear features adds little discriminative value.

Our AUC of 0.927 compares favorably with, and in several cases exceeds, previously published OCTA-based supervised-learning models for T2DM classification. Fernández-Espinosa et al. reported AUCs of 0.77–0.86 for random-forest and gradient-boosting classifiers trained on OCTA + clinical data in a cohort of 372 patients [45]. Studies using deep-learning approaches on OCTA images directly have reported AUCs in the 0.85–0.93 range, but at the cost of interpretability [46,47]. The SHAP-based ranking directly addresses this interpretability challenge: FD-300, CC flow ratio, DCP parafoveal VD, and retinal-thickness subfields emerge as the leading features, each corresponding to a biologically meaningful compartment rather than a latent deep-learning representation. This combination of screening-grade performance and feature-level interpretability is particularly attractive for clinical deployment because it enables a model output (the probability of preclinical DR) to be accompanied by an explanation of which biomarkers drove the classification, thereby supporting clinician review and patient communication. SHAP-based DR prediction frameworks have recently begun to appear in clinical decision-support contexts [48,49], and the present work extends this approach to commercial-grade automated OCTA output without requiring custom image processing.

### 4.10. Strengths, Limitations, and Clinical Implications

The principal strengths of the present study are the following: (i) the comprehensive 68-parameter OCTA panel covering all commercial AngioAnalytic output compartments, (ii) the application of FDR correction across the full panel rather than the small, preselected subsets typical of earlier work, (iii) the use of nonlinear ensemble classifiers with explicit comparison to conventional and clinically interpretable reduced panels, and (iv) SHAP-based model interpretation that yields a biomarker ranking suitable for clinical use. The sample size (221 eyes from 113 participants) provides substantial statistical power for all principal comparisons and enables meaningful subgroup analyses.

Several limitations should be acknowledged. First, the study is cross-sectional, so the temporal sequence of multi-compartment changes cannot be inferred directly; longitudinal follow-up of the same cohort would be needed to determine which biomarkers change first and which best predict progression to clinical DR. Second, both eyes of each participant were imaged; inter-eye dependence was addressed through patient-grouped cross-validation, cluster-robust standard errors, and a one-eye-per-participant analysis (Section 2.6). Generalized estimating equations and random-intercept mixed models are not applicable here because the outcome is constant within each participant, but the approaches used provide appropriate adjustment for this design. Third, the OCTA device used (Optovue RTVue Avanti) is a spectral-domain platform with limited projection-artifact removal, which affects interpretation of the outer-retinal flow signal (Section 4.5) and likely also affects quantitative DCP metrics at the margins [31]. Swept-source systems with advanced projection-resolved algorithms [32] may provide additional compartment-specific resolution. Fourth, disease duration in the T2DM group was ascertained from self-report and clinical records rather than from biomarkers of cumulative glycemic exposure (such as skin autofluorescence), which would more precisely operationalize the metabolic-memory framework. Fifth, we did not include continuous glucose monitoring-derived metrics such as glycemic variability or time-in-range, which recent work in T1DM cohorts suggests may add complementary information [44]. Sixth, the single-center design limits immediate generalizability to populations with different ethnic, metabolic, or ocular backgrounds; external validation in an independent cohort is the most important next step. Finally, the XGBoost model was internally cross-validated rather than trained on a separate validation cohort. While patient-grouped cross-validation with an outlier-sensitivity analysis provides strong internal robustness, external validation is required before clinical deployment. Finally, the cohort did not include a comparison group of patients with clinically manifest diabetic retinopathy. The present analysis, therefore, characterizes the preclinical T2DM-versus-control contrast rather than the staging of established disease, and validation against an independent cohort that also includes diabetic-retinopathy-positive eyes is an important direction for future work. Future work should address these limitations through longitudinal follow-up, replication with swept-source OCTA, incorporation of CGM-derived metrics, and multi-center external validation.

The present results have three concrete clinical implications. First, OCTA-derived biomarkers—particularly FD-300, parafoveal DCP vessel density, choriocapillaris flow, and FAZ circularity—are detectable well before any fundoscopic evidence of DR. When combined in a supervised nonlinear model, they achieve screening-grade classification performance on a standard commercial platform without custom image processing. This supports the emerging view that OCTA should be considered for inclusion in routine diabetic eye examinations, particularly in patients with long-standing disease or comorbid cardiometabolic risk factors [9,50]. Second, the near-complete decoupling of the OCTA signature from current HbA1c means that good contemporary glycemic control does not preclude substantial preclinical retinal microvasculopathy; OCTA screening should therefore not be deferred in well-controlled T2DM. Third, the selective further compromise of the choroidal compartment in T2DM with concomitant hypertension highlights the importance of integrated cardiometabolic management and suggests that patients with combined diabetes and hypertension may warrant more intensive ophthalmic follow-up than those with isolated diabetes. Prospective validation of OCTA-based screening thresholds and intervention strategies, and evaluation of whether early OCTA detection translates into improved long-term visual outcomes, are the natural next steps.

## 5. Conclusions

In a prospective cohort of T2DM eyes without clinical retinopathy, a comprehensive 68-parameter OCTA analysis identified widespread preclinical microvascular, structural, and choroidal changes. The deep capillary plexus vessel density was the most severely affected compartment. FD-300 emerged as the single most influential biomarker in the classifier, and the retinal-thickness map revealed a topographically heterogeneous pattern consistent with early neurodegeneration and subclinical fluid redistribution. The OCTA signature was robust to adjustment for systemic confounders, effectively decoupled from current HbA1c, and statistically indistinguishable between well-controlled and poorly controlled T2DM—a pattern consistent with metabolic memory. A supervised XGBoost model trained on the full 68-parameter panel achieved a patient-grouped cross-validated AUC of approximately 0.91, and SHAP-based interpretability localized the classifier’s decisions to biologically meaningful compartments. Together, these findings support the use of OCTA as a quantitative screening modality for preclinical DR in T2DM, independent of current glycemic control, and motivate prospective, longitudinal, multicenter validation of the proposed biomarker panel.

## Figures and Tables

**Table 1:** Baseline demographic and clinical characteristics.

Parameter	Control (*n* = 66)	T2DM (*n* = 155)	*p*-Value	Test
Age (years), mean ± SD	55.3 ± 12.3	58.7 ± 13.0	0.031	Mann–Whitney U
Sex (M/F)	30/36	80/75	0.490	Chi-squared
HbA1c (%), mean ± SD	5.05 ± 0.56	7.96 ± 2.36	<0.001	Mann–Whitney U
HbA1c range (%)	4.1–6.1	5.2–16.7	–	–
Arterial hypertension, n (%)	16 (24.2%)	85 (54.8%)	<0.001	Chi-squared
Hyperlipidemia, n (%)	4 (6.1%)	28 (18.1%)	0.035	Chi-squared

Data from 221 eyes after outlier screening: 155 T2DM eyes from 80 patients and 66 control eyes from 33 volunteers. Between-group comparisons: Mann–Whitney U for continuous variables, Pearson chi-squared for categorical. HbA1c = glycated hemoglobin.

**Table 2:** Principal OCTA biomarker comparisons between T2DM and Control.

Parameter	Control Median [Q1, Q3]	T2DM Median [Q1, Q3]	rb	*p*	q (FDR)
**Foveal Density (FD-300)**					
FD-300 (%)	51.86 [48.52, 54.56]	47.55 [42.30, 49.50]	−0.57	<0.001	**1.01 × 10^−10^**
Foveal Avascular Zone					
FAZ area (mm^2^)	0.30 [0.24, 0.37]	0.27 [0.23, 0.34]	−0.17	0.048	**0.070**
FAZ perimeter (mm)	2.24 [1.98, 2.46]	2.15 [1.89, 2.40]	−0.10	0.248	**0.277**
FAZ Circularity Index	0.80 [0.75, 0.82]	0.76 [0.69, 0.81]	−0.24	0.005	**0.010**
FAZ Acircularity Index	0.20 [0.18, 0.25]	0.24 [0.19, 0.31]	+0.24	0.005	**0.010**
SCP Vessel Density					
SCP VD–Whole image (%)	47.60 [45.70, 49.00]	43.30 [39.05, 46.80]	−0.52	<0.001	**3.62 × 10^−9^**
SCP VD–Parafovea (%)	51.10 [49.10, 52.55]	45.70 [42.10, 49.95]	−0.57	<0.001	**1.01 × 10^−10^**
DCP Vessel Density					
DCP VD–Whole image (%)	54.45 [52.00, 55.80]	48.10 [45.10, 51.75]	−0.66	<0.001	**2.50 × 10^−13^**
DCP VD–Parafovea (%)	56.30 [54.40, 57.98]	50.20 [46.85, 54.05]	−0.64	<0.001	**7.57 × 10^−13^**
Retinal Thickness (ETDRS map)					
Retinal thickness–Fovea (µm)	253.00 [243.00, 265.00]	265.00 [248.00, 287.00]	+0.26	0.002	**0.005**
Retinal thickness–Parafovea (µm)	316.00 [306.25, 326.75]	311.00 [300.00, 323.00]	−0.18	0.038	**0.057**
Outer Retina					
OR–Flow area (mm^2^)	1.11 [0.95, 1.40]	1.68 [1.21, 2.51]	+0.39	<0.001	**1.34 × 10^−5^**
OR–Flow ratio	0.16 [0.14, 0.20]	0.24 [0.17, 0.36]	+0.39	<0.001	**1.30 × 10^−5^**
Choriocapillaris					
CC–Flow area (mm^2^)	4.80 [4.63, 4.94]	4.70 [4.44, 4.92]	−0.22	0.011	**0.021**
CC–Flow ratio	0.68 [0.66, 0.70]	0.66 [0.63, 0.69]	−0.25	0.004	**0.009**

Values are median [Q1, Q3]. Between-group comparisons: Mann–Whitney U. rb = rank-biserial correlation (negative = T2DM < Control). q = Benjamini–Hochberg FDR-adjusted *p*-value across all 68 OCTA parameters (see Supplementary Table S1 for full panel). Parameters in bold reach FDR significance (q < 0.05). SCP = superficial capillary plexus; DCP = deep capillary plexus; VD = vessel density; FAZ = foveal avascular zone; FD-300 = foveal density (vessel density in 300 µm annulus around FAZ); OR = outer retina; CC = choriocapillaris.

**Table 3:** Machine-learning classifier performance (revised).

Model	# Features	Standard 10-Fold CV	Patient-Grouped 10-Fold CV
Conventional VD (SCP + DCP whole image)	2	0.848 ± 0.076	0.865 ± 0.072
FD-300 alone	1	0.786 ± 0.144	0.792 ± 0.074
Integrative 5-feature panel	5	0.873 ± 0.084	0.877 ± 0.053
Logistic Regression—all OCTA	68	0.856 ± 0.085	0.864 ± 0.065
Random Forest–all OCTA	68	0.909 ± 0.050	0.909 ± 0.088
XGBoost–all OCTA (primary model)	68	0.927 ± 0.049	0.927 ± 0.085

Values are mean ± SD of the area under the ROC curve across cross-validation folds. Patient-grouped cross-validation (StratifiedGroupKFold) assigns both eyes of a participant to the same fold and is the primary scheme. For the primary XGBoost model the pooled out-of-fold AUC under patient grouping was 0.909 (0.88–0.93 across five random fold assignments). The conventional two-feature panel and the FD-300-alone, integrative five-feature, and 68-feature logistic-regression rows are logistic-regression models; the random forest and XGBoost rows use the full 68-feature panel. HbA1c was excluded from all models.

**Table 4:** Multivariable logistic regression for type 2 diabetes status (cluster-robust inference).

Variable	Coefficient (β)	*p*-Value	Odds Ratio	95% CI
FD-300 (per %)	−0.278	<0.001	0.758	0.673–0.852
Arterial hypertension (HTA)	+1.375	0.014	3.955	1.317–11.882
Hyperlipidemia (HLP)	+0.852	0.28	2.345	0.506–10.862
Age (years)	−0.010	0.59	0.990	0.953–1.028

Eye-level multivariable logistic regression (n = 221 eyes; 114 participants). All coefficients, odds ratios, 95% confidence intervals, and *p*-values are from a single logistic model fitted with cluster-robust (Huber–White sandwich) standard errors, clustered at the participant level, to account for the inclusion of both eyes per participant. FD-300 odds ratio is expressed per 1% increase; age per year.

## Data Availability

The authors confirm that the data supporting the findings of this study are available within the article.

## Supplementary Materials

The following supporting information can be downloaded at: https://www.mdpi.com/article/10.3390/medicina62061153/s1, Table S1. Full statistical results for all 68 OCTA parameters (between-group comparisons with FDR correction). Table S2. Top 15 FDR-significant OCTA parameters ranked by |rb|. Table S3. Univariate AUC for all 68 OCTA parameters. Table S4. Top 30 Spearman correlations between OCTA parameters within the T2DM cohort. Table S5. Top 25 HbA1c–OCTA Spearman correlations within T2DM. Table S6. HTA stratification within the T2DM cohort. Table S7. Outlier screening sensitivity analysis. Table S8. Pre-specified logistic-regression panels (A–H) performance. Table S9. Full SHAP top-20 feature ranking for the XGBoost classifier. Figure S1. Volcano plot of all 68 OCTA parameters: rank-biserial effect size (T2DM versus Control) on the horizontal axis versus −log_10_(FDR-adjusted q-value) on the vertical axis, colour-coded by biomarker family. Figure S2. Univariate ROC-AUC bar chart for all 68 OCTA parameters, ranked by descending discriminative power for the T2DM-versus-Control task. Figure S3. Relationship between FD-300 and SCP parafoveal vessel density, stratified by diagnostic group. Figure S4. Receiver-operating-characteristic (ROC) curves for the eight pre-specified logistic-regression panels (A–H) listed in Table S8, under patient-grouped cross-validation. Figure S5. SHAP dependence plots for the top six features identified in the XGBoost classifier.
