# Supplementary material for: Foveal Density and Multi-Domain OCTA Biomarkers May Help Identify Preclinical Diabetic Microvasculopathy in Type 2 Diabetes Mellitus
Source: Medicina (Kaunas). 2026 Jun 13;62(6):1153. doi: 10.3390/medicina62061153 (PMC13303164; doi:10.3390/medicina62061153)
Supplement: Supplementary file 1 [file medicina-62-01153-s001.zip › medicina-4315476-supplementary.pdf]

Supplementary Materials

Contents

Table S1. Full statistical results for all 68 OCTA parameters (between-group comparisons with FDR correction).

Table S2. Top 15 FDR-significant OCTA parameters ranked by $|r_b|$.

Table S3. Univariate AUC for all 68 OCTA parameters.

Table S4. Top 30 Spearman correlations between OCTA parameters within the T2DM cohort.

Table S5. Top 25 HbA1c–OCTA Spearman correlations within T2DM.

Table S6. HTA stratification within the T2DM cohort.

Table S7. Outlier screening sensitivity analysis.

Table S8. Pre-specified logistic-regression panels (A–H) performance.

Table S9. Full SHAP top-20 feature ranking for the XGBoost classifier.

Figure S1. Volcano plot of all 68 OCTA parameters: rank-biserial effect size (T2DM versus Control) on the horizontal axis versus $-\log_{10}$(FDR-adjusted *q*-value) on the vertical axis, colour-coded by biomarker family.

Figure S2. Univariate ROC-AUC bar chart for all 68 OCTA parameters, ranked by descending discriminative power for the T2DM-versus-Control task.

Figure S3. Relationship between FD-300 and SCP parafoveal vessel density, stratified by diagnostic group.

Figure S4. Receiver-operating-characteristic (ROC) curves for the eight pre-specified logistic-regression panels (A–H) listed in Table S8, under patient-grouped cross-validation.

Figure S5. SHAP dependence plots for the top six features identified in the XGBoost classifier.

Table S1. Full between-group comparison of all 68 OCTA parameters.

Parameter	Control median [Q1, Q3]	T2DM median [Q1, Q3]	r_{b}	p	q (FDR)
**DCP VD – Inferior hemi**	54.10 [51.63, 55.58]	48.20 [44.35, 51.25]	−0.663	6.72e−15	**2.50e−13**
**DCP VD – Whole image**	54.45 [52.00, 55.80]	48.10 [45.10, 51.75]	−0.662	7.36e−15	**2.50e−13**
**DCP VD – Parafovea I-hemi**	55.75 [54.52, 57.80]	50.20 [46.25, 53.80]	−0.649	2.39e−14	**5.42e−13**
**DCP VD – Parafovea**	56.30 [54.40, 57.98]	50.20 [46.85, 54.05]	−0.642	4.45e−14	**7.57e−13**
**DCP VD – Superior hemi**	54.50 [52.13, 56.50]	48.40 [45.30, 52.10]	−0.638	6.37e−14	**8.32e−13**
**DCP VD – Parafovea Temporal**	55.75 [54.33, 57.70]	49.70 [46.25, 53.60]	−0.636	7.34e−14	**8.32e−13**
**DCP VD – Parafovea S-hemi**	56.00 [54.23, 58.30]	50.60 [47.45, 54.60]	−0.611	7.05e−13	**6.85e−12**
**DCP VD – Parafovea Nasal**	55.95 [54.92, 57.40]	50.30 [47.10, 54.80]	−0.601	1.53e−12	**1.22e−11**
**DCP VD – Parafovea Inferior**	56.05 [54.40, 57.98]	49.50 [45.75, 53.50]	−0.601	1.61e−12	**1.22e−11**
**DCP VD – Parafovea Superior**	56.80 [54.23, 58.55]	50.70 [47.80, 55.05]	−0.582	7.77e−12	**5.28e−11**
**SCP VD – Parafovea**	51.10 [49.10, 52.55]	45.70 [42.10, 49.95]	−0.573	1.68e−11	**1.01e−10**
**FD-300 (foveal density %)**	51.86 [48.52, 54.56]	47.55 [42.30, 49.50]	−0.572	1.78e−11	**1.01e−10**
**SCP VD – Parafovea Inferior**	52.60 [49.60, 54.20]	47.10 [43.30, 51.00]	−0.550	1.01e−10	**5.28e−10**
**SCP VD – Whole image**	47.60 [45.70, 49.00]	43.30 [39.05, 46.80]	−0.524	7.45e−10	**3.62e−9**
**SCP VD – Parafovea S-hemi**	50.40 [48.30, 52.50]	45.90 [41.50, 49.60]	−0.513	1.58e−9	**7.18e−9**
**SCP VD – Parafovea I-hemi**	51.20 [48.83, 52.60]	46.20 [42.30, 49.75]	−0.511	1.87e−9	**7.96e−9**
**SCP VD – Parafovea Nasal**	50.40 [47.95, 51.65]	45.60 [41.50, 49.50]	−0.510	2.03e−9	**8.11e−9**
**SCP VD – Superior hemi**	47.25 [45.38, 49.15]	43.60 [38.30, 46.70]	−0.504	3.07e−9	**1.16e−8**
**SCP VD – Inferior hemi**	47.25 [45.52, 48.90]	43.00 [38.20, 46.70]	−0.503	3.36e−9	**1.20e−8**
**SCP VD – Parafovea Superior**	51.55 [49.78, 53.58]	46.70 [42.85, 51.10]	−0.495	5.78e−9	**1.96e−8**
**SCP VD – Parafovea Temporal**	48.95 [46.83, 50.10]	45.30 [40.40, 48.40]	−0.463	5.36e−8	**1.73e−7**
**OR – Flow ratio**	0.16 [0.14, 0.20]	0.24 [0.17, 0.36]	+0.391	4.21e−6	**1.30e−5**
**OR – Flow area (mm²)**	1.11 [0.95, 1.40]	1.68 [1.21, 2.51]	+0.390	4.52e−6	**1.34e−5**
**Retinal thickness – Parafovea Superior**	323.50 [313.00, 332.00]	313.00 [300.00, 333.00]	−0.280	9.81e−4	**0.003**
**DCP Thickness – Parafovea**	325.00 [321.00, 337.75]	321.00 [312.50, 334.00]	−0.265	0.002	**0.005**
**Retinal thickness – Fovea**	253.00 [243.00, 265.00]	265.00 [248.00, 287.00]	+0.262	0.002	**0.005**
**SCP Thickness – Fovea**	251.50 [242.00, 266.75]	261.00 [247.00, 276.00]	+0.257	0.002	**0.006**
**CC – Flow ratio**	0.68 [0.66, 0.70]	0.66 [0.63, 0.69]	−0.248	0.004	**0.009**
**FAZ Circularity Index**	0.80 [0.75, 0.82]	0.76 [0.69, 0.81]	−0.242	0.005	**0.010**
**FAZ Acircularity Index**	0.20 [0.18, 0.25]	0.24 [0.19, 0.31]	+0.242	0.005	**0.010**
**SCP Thickness – Parafovea Superior**	329.50 [326.00, 340.00]	325.00 [313.50, 340.00]	−0.237	0.005	**0.012**
**Retinal thickness – Perifovea Superior**	293.00 [285.00, 300.00]	287.00 [274.50, 298.00]	−0.225	0.008	**0.017**
**DCP Thickness – Parafovea Superior**	329.00 [326.00, 340.00]	325.00 [313.50, 340.00]	−0.222	0.009	**0.019**
**CC – Flow area (mm²)**	4.80 [4.63, 4.94]	4.70 [4.44, 4.92]	−0.218	0.011	**0.021**
**DCP Thickness – Parafovea Inferior**	327.00 [320.00, 340.00]	322.00 [311.00, 334.00]	−0.208	0.015	**0.028**
**DCP Thickness – Parafovea I-hemi**	324.50 [318.00, 336.75]	319.00 [312.00, 332.00]	−0.204	0.017	**0.031**

Parameter	Control median [Q1, Q3]	T2DM median [Q1, Q3]	r_{b}	p	q (FDR)
**DCP Thickness – Parafovea S-hemi**	325.00 [321.25, 337.75]	323.00 [311.00, 336.50]	−0.202	0.017	**0.032**
**SCP Thickness – Parafovea Inferior**	327.00 [320.25, 339.25]	321.00 [308.50, 334.00]	−0.201	0.018	**0.032**
**DCP Thickness – Fovea**	252.50 [242.00, 265.50]	259.00 [247.00, 274.00]	+0.200	0.018	**0.032**
**SCP Thickness – Parafovea S-hemi**	326.00 [322.25, 336.75]	323.00 [311.50, 337.00]	−0.200	0.019	**0.032**
**SCP Thickness – Parafovea**	324.50 [321.00, 336.75]	322.00 [312.00, 334.50]	−0.198	0.020	**0.033**
**SCP Thickness – Parafovea I-hemi**	325.00 [319.25, 336.75]	321.00 [311.50, 332.00]	−0.193	0.023	**0.038**
DCP Thickness – Parafovea Temporal	319.50 [312.00, 328.75]	314.00 [305.00, 327.00]	−0.179	0.035	0.055
DCP Thickness – Whole image	314.50 [310.25, 326.00]	312.00 [303.00, 324.00]	−0.178	0.037	0.057
Retinal thickness – Parafovea	316.00 [306.25, 326.75]	311.00 [300.00, 323.00]	−0.176	0.038	0.057
DCP Thickness – Superior hemi	315.00 [312.25, 327.00]	313.00 [303.00, 326.00]	−0.176	0.039	0.057
FAZ area (mm²)	0.30 [0.24, 0.37]	0.27 [0.23, 0.34]	−0.168	0.048	0.070
Retinal thickness – Parafovea S-hemi	317.50 [308.00, 325.00]	312.00 [300.00, 327.50]	−0.161	0.058	0.082
Retinal thickness – Perifovea S-hemi	290.50 [285.50, 299.75]	289.00 [275.00, 298.00]	−0.158	0.063	0.088
Retinal thickness – Perifovea Inferior	282.00 [275.00, 294.50]	277.00 [264.00, 293.00]	−0.152	0.074	0.101
SCP Thickness – Superior hemi	315.00 [312.25, 326.75]	313.00 [303.00, 328.00]	−0.145	0.087	0.116
SCP Thickness – Inferior hemi	314.00 [308.00, 326.00]	312.00 [302.50, 325.00]	−0.127	0.134	0.175
Retinal thickness – Parafovea Nasal	320.00 [312.00, 329.00]	316.00 [302.00, 331.50]	−0.125	0.143	0.183
DCP Thickness – Parafovea Nasal	327.50 [322.00, 342.75]	327.00 [312.50, 339.00]	−0.122	0.151	0.191
DCP Thickness – Inferior hemi	314.00 [307.25, 327.75]	312.00 [303.00, 325.00]	−0.120	0.157	0.194
SCP Thickness – Whole image	314.00 [311.00, 325.00]	314.00 [303.00, 326.00]	−0.117	0.171	0.206
SCP Thickness – Parafovea Nasal	326.50 [322.25, 341.75]	328.00 [311.50, 340.50]	−0.116	0.173	0.206
Retinal thickness – Perifovea I-hemi	284.50 [277.25, 294.75]	282.00 [271.00, 292.00]	−0.114	0.182	0.213
Retinal thickness – Perifovea	288.00 [280.00, 297.00]	287.00 [274.00, 297.50]	−0.106	0.214	0.246
SCP Thickness – Parafovea Temporal	316.50 [312.25, 325.75]	314.00 [305.00, 328.00]	−0.101	0.235	0.266
FAZ perimeter (mm)	2.24 [1.98, 2.46]	2.15 [1.89, 2.40]	−0.098	0.248	0.277
Retinal thickness – Parafovea Inferior	314.00 [306.25, 324.75]	311.00 [298.50, 324.00]	−0.092	0.282	0.309
Retinal thickness – Parafovea I-hemi	313.50 [305.00, 323.50]	312.00 [298.50, 324.00]	−0.075	0.376	0.406
Retinal thickness – Perifovea Nasal	299.00 [289.25, 308.00]	298.00 [285.00, 311.00]	−0.072	0.399	0.424
SCP VD – Fovea	16.05 [11.93, 20.68]	16.60 [12.80, 22.95]	+0.054	0.525	0.549
DCP VD – Fovea	32.40 [27.40, 37.48]	31.30 [26.80, 36.80]	−0.050	0.559	0.576
Retinal thickness – Parafovea Temporal	307.50 [301.25, 318.00]	306.00 [298.00, 318.00]	−0.040	0.642	0.652
Retinal thickness – Perifovea Temporal	280.00 [272.25, 287.00]	279.00 [268.00, 290.00]	+0.032	0.705	0.705

Full panel of 68 OCTA parameters. Sorted by FDR-adjusted q-value. Parameters reaching q < 0.05 are bold. r_{b} = rank-biserial correlation (negative = T2DM < Control). 42 of 68 parameters reach FDR significance.

Table S2. Top 15 FDR-significant OCTA parameters ranked by |r_{b}|.

Rank	OCTA parameter	r_{b}	p	q (FDR)
**1**	**DCP VD – Inferior hemi**	−0.663	< 0.001	**2.50e−13**
**2**	**DCP VD – Whole image**	−0.662	< 0.001	**2.50e−13**
**3**	**DCP VD – Parafovea I-hemi**	−0.649	< 0.001	**5.42e−13**
**4**	**DCP VD – Parafovea**	−0.642	< 0.001	**7.57e−13**
**5**	**DCP VD – Superior hemi**	−0.638	< 0.001	**8.32e−13**
**6**	**DCP VD – Parafovea Temporal**	−0.636	< 0.001	**8.32e−13**
**7**	**DCP VD – Parafovea S-hemi**	−0.611	< 0.001	**6.85e−12**
**8**	**DCP VD – Parafovea Nasal**	−0.601	< 0.001	**1.22e−11**
**9**	**DCP VD – Parafovea Inferior**	−0.601	< 0.001	**1.22e−11**
**10**	**DCP VD – Parafovea Superior**	−0.582	< 0.001	**5.28e−11**
**11**	**SCP VD – Parafovea**	−0.573	< 0.001	**1.01e−10**
**12**	**FD-300 (foveal density %)**	−0.572	< 0.001	**1.01e−10**
**13**	**SCP VD – Parafovea Inferior**	−0.550	< 0.001	**5.28e−10**
**14**	**SCP VD – Whole image**	−0.524	< 0.001	**3.62e−9**
**15**	**SCP VD – Parafovea S-hemi**	−0.513	< 0.001	**7.18e−9**

All 15 parameters reach FDR significance at q < 0.05. Negative r_{b} = T2DM < Control; positive r_{b} = T2DM > Control. The top ten positions are occupied exclusively by members of the DCP vessel-density family.

Table S3. Univariate AUC for all 68 OCTA parameters.

Rank	OCTA parameter	Univariate AUC
1	DCP VD – Inferior hemi	0.831
2	DCP VD – Whole image	0.831
3	DCP VD – Parafovea I-hemi	0.824
4	DCP VD – Parafovea	0.821
5	DCP VD – Superior hemi	0.819
6	DCP VD – Parafovea Temporal	0.818
7	DCP VD – Parafovea S-hemi	0.805
8	DCP VD – Parafovea Nasal	0.801
9	DCP VD – Parafovea Inferior	0.800
10	DCP VD – Parafovea Superior	0.791
11	SCP VD – Parafovea	0.786
12	FD-300 (foveal density %)	0.786
13	SCP VD – Parafovea Inferior	0.775
14	SCP VD – Whole image	0.762

Rank	OCTA parameter	Univariate AUC
15	SCP VD – Parafovea S-hemi	0.757
16	SCP VD – Parafovea I-hemi	0.756
17	SCP VD – Parafovea Nasal	0.755
18	SCP VD – Superior hemi	0.752
19	SCP VD – Inferior hemi	0.751
20	SCP VD – Parafovea Superior	0.748
21	SCP VD – Parafovea Temporal	0.731
22	OR – Flow ratio	0.696
23	OR – Flow area (mm^2)	0.695
24	Retinal thickness – Parafovea Superior	0.640
25	DCP Thickness – Parafovea	0.633
26	Retinal thickness – Fovea	0.631
27	SCP Thickness – Fovea	0.629
28	CC – Flow ratio	0.624
29	FAZ Circularity Index	0.621
30	FAZ Acircularity Index	0.621
31	SCP Thickness – Parafovea Superior	0.618
32	Retinal thickness – Perifovea Superior	0.612
33	DCP Thickness – Parafovea Superior	0.611
34	CC – Flow area (mm^2)	0.609
35	DCP Thickness – Parafovea Inferior	0.604
36	DCP Thickness – Parafovea I-hemi	0.602
37	DCP Thickness – Parafovea S-hemi	0.601
38	SCP Thickness – Parafovea Inferior	0.600
39	DCP Thickness – Fovea	0.600
40	SCP Thickness – Parafovea S-hemi	0.600
41	SCP Thickness – Parafovea	0.599
42	SCP Thickness – Parafovea I-hemi	0.596
43	DCP Thickness – Parafovea Temporal	0.590
44	DCP Thickness – Whole image	0.589
45	Retinal thickness – Parafovea	0.588
46	DCP Thickness – Superior hemi	0.588
47	FAZ area (mm^2)	0.584
48	Retinal thickness – Parafovea S-hemi	0.581
49	Retinal thickness – Perifovea S-hemi	0.579
50	Retinal thickness – Perifovea Inferior	0.576
51	SCP Thickness – Superior hemi	0.573
52	SCP Thickness – Inferior hemi	0.564

Rank	OCTA parameter	Univariate AUC
53	Retinal thickness – Parafovea Nasal	0.562
54	DCP Thickness – Parafovea Nasal	0.561
55	DCP Thickness – Inferior hemi	0.560
56	SCP Thickness – Whole image	0.558
57	SCP Thickness – Parafovea Nasal	0.558
58	Retinal thickness – Perifovea I-hemi	0.557
59	Retinal thickness – Perifovea	0.553
60	SCP Thickness – Parafovea Temporal	0.551
61	FAZ perimeter (mm)	0.549
62	Retinal thickness – Parafovea Inferior	0.546
63	Retinal thickness – Parafovea I-hemi	0.538
64	Retinal thickness – Perifovea Nasal	0.536
65	SCP VD – Fovea	0.527
66	DCP VD – Fovea	0.525
67	Retinal thickness – Parafovea Temporal	0.520
68	Retinal thickness – Perifovea Temporal	0.516

Ranked by univariate AUC for T2DM vs Control discrimination. Parameters with AUC < 0.55 carry negligible discriminative value individually but may contribute to multivariable models through interaction effects.

Table S4. Top 30 Spearman correlations between pairs of OCTA parameters within T2DM.

Parameter A	Parameter B	Spearman ρ	q (FDR)
SCP VD – Whole image	SCP VD – Parafovea	+0.93	< 1e−40
DCP VD – Whole image	DCP VD – Parafovea	+0.92	< 1e−40
DCP VD – Superior hemi	DCP VD – Inferior hemi	+0.88	< 1e−35
SCP VD – Superior hemi	SCP VD – Inferior hemi	+0.85	< 1e−32
SCP VD – Parafovea	FD-300	+0.71	1.3e−23
SCP VD – Whole image	FD-300	+0.64	3.7e−18
SCP VD – Whole image	DCP VD – Whole image	+0.42	2.1e−8
SCP VD – Parafovea	DCP VD – Parafovea	+0.43	8.0e−9
SCP VD – Parafovea	FAZ Circularity	+0.30	5.2e−4
FD-300	FAZ Circularity	+0.30	6.9e−4
CC – Flow area	CC – Flow ratio	+0.95	< 1e−40
OR – Flow area	OR – Flow ratio	+0.97	< 1e−40
SCP Thickness – Parafovea	Retinal thickness – Parafovea	+0.74	7.2e−26
DCP Thickness – Parafovea	Retinal thickness – Parafovea	+0.68	1.1e−21
FAZ area	FAZ perimeter	+0.95	< 1e−40
FAZ area	FAZ Circularity	−0.38	1.8e−6

Parameter A	Parameter B	Spearman ρ	q (FDR)
FAZ area	FD-300	−0.31	3.2e−4
DCP VD – Parafovea	OR – Flow area	−0.27	1.6e−3
SCP VD – Parafovea	OR – Flow area	−0.33	3.1e−5
FD-300	OR – Flow area	−0.27	1.2e−3
DCP VD – Whole image	DCP VD – Parafovea Temporal	+0.89	< 1e−35
DCP VD – Parafovea	DCP VD – Parafovea Superior	+0.87	< 1e−34
DCP VD – Parafovea	DCP VD – Parafovea Nasal	+0.85	< 1e−32
DCP VD – Parafovea	DCP VD – Parafovea Inferior	+0.86	< 1e−33
SCP VD – Whole image	SCP VD – Parafovea Temporal	+0.87	< 1e−34
Retinal thickness – Fovea	FAZ area	−0.39	6.8e−7
Retinal thickness – Fovea	CC – Flow area	+0.24	3.1e−3
Retinal thickness – Parafovea	DCP Thickness – Parafovea	+0.68	1.1e−21
DCP VD – Parafovea I-hemi	DCP VD – Parafovea S-hemi	+0.82	< 1e−28
SCP VD – Parafovea I-hemi	SCP VD – Parafovea S-hemi	+0.83	< 1e−29

Top 30 pairwise correlations by |ρ| within the T2DM cohort (n = 155). All listed pairs reach FDR significance. Three structural groups are visible: (i) within-family correlations (|ρ| > 0.85), (ii) cross-family vascular (|ρ| 0.42–0.71), (iii) structural-vascular (|ρ| 0.68–0.74).

Table S5. Top 25 HbA1c–OCTA Spearman correlations within T2DM (n = 155).

OCTA parameter	Spearman ρ with HbA1c	p-value	q (FDR)
Retinal thickness – Parafovea	−0.166	0.039	0.984
OR – Flow area	+0.124	0.123	0.984
OR – Flow ratio	+0.121	0.134	0.984
DCP VD – Parafovea S-hemi	+0.108	0.179	0.984
DCP VD – Parafovea Superior	+0.086	0.289	0.984
DCP VD – Parafovea Nasal	+0.084	0.299	0.984
FAZ perimeter	+0.050	0.540	0.984
DCP VD – Parafovea I-hemi	+0.046	0.567	0.984
DCP VD – Parafovea	+0.043	0.598	0.984
SCP VD – Parafovea Inferior	+0.040	0.624	0.984
SCP VD – Parafovea Temporal	−0.034	0.674	0.984
SCP VD – Parafovea S-hemi	+0.034	0.674	0.984
DCP VD – Whole image	+0.033	0.679	0.984
SCP VD – Whole image	+0.033	0.681	0.984
DCP VD – Parafovea Inferior	+0.032	0.694	0.984
SCP VD – Parafovea Superior	+0.031	0.703	0.984
CC – Flow ratio	−0.031	0.704	0.984
FD-300	+0.026	0.750	0.984

OCTA parameter	Spearman ρ with HbA1c	p-value	q (FDR)
FAZ area	+0.024	0.763	0.984
SCP VD – Parafovea Nasal	+0.023	0.775	0.984
FAZ Circularity	+0.019	0.813	0.984
SCP VD – Parafovea	+0.013	0.869	0.984
CC – Flow area	−0.010	0.899	0.984
DCP VD – Parafovea Temporal	−0.006	0.945	0.984
Retinal thickness – Fovea	−0.005	0.955	0.984

Top 25 by |ρ| within T2DM. FDR correction applied across all 68 correlations. No correlation reaches FDR significance (all $q \geq 0.98$). Even the largest raw correlations (retinal thickness parafovea $\rho = -0.17$; OR flow $\rho = +0.12$) have magnitudes that account for less than 3% of the variance in the OCTA biomarker.

Table S6. HTA stratification within the T2DM cohort.

Parameter	Control (n = 66)	T2DM − HTA (n = 70)	T2DM + HTA (n = 85)	p (−HTA vs +HTA)	q (FDR)
FD-300 (%)	51.4 ± 4.0	45.5 ± 5.6	46.1 ± 5.8	0.665	0.721
**FAZ area (mm²)**	0.31 ± 0.09	0.27 ± 0.09	0.31 ± 0.15	**0.046**	**0.100**
FAZ Circularity	0.79 ± 0.07	0.77 ± 0.26	0.75 ± 0.11	0.441	0.573
SCP VD – Whole image	46.9 ± 3.9	41.7 ± 7.2	42.1 ± 6.7	0.805	0.805
SCP VD – Parafovea	50.6 ± 4.0	44.9 ± 6.1	45.3 ± 5.7	0.731	0.791
DCP VD – Whole image	53.7 ± 3.0	48.2 ± 5.1	48.3 ± 4.9	0.524	0.618
DCP VD – Parafovea	55.8 ± 2.9	50.2 ± 5.5	50.3 ± 5.5	0.585	0.634
OR – Flow area (mm²)	1.38 ± 0.79	1.79 ± 0.88	2.20 ± 2.23	0.170	0.277
OR – Flow ratio	0.20 ± 0.11	0.25 ± 0.12	0.31 ± 0.31	0.168	0.277
**CC – Flow area (mm²)**	4.79 ± 0.25	4.73 ± 0.33	4.58 ± 0.37	**0.008**	**0.026**
**CC – Flow ratio**	0.68 ± 0.04	0.66 ± 0.09	0.63 ± 0.10	**0.008**	**0.026**
**Retinal thickness – Fovea (μm)**	255 ± 21	275 ± 35	261 ± 22	**0.035**	**0.091**
**Retinal thickness – Parafovea (μm)**	316 ± 14	317 ± 19	306 ± 24	**0.005**	**0.033**

Values are mean ± SD. p-values from Mann–Whitney U comparing the two T2DM subgroups (−HTA vs +HTA) directly. q-values reflect Benjamini–Hochberg FDR correction across the 13 listed parameters. Parameters reaching $q < 0.05$ (in bold) indicate selective HTA-associated effects primarily on the choroidal compartment and retinal-thickness measures; principal diabetes-driven biomarkers (FD-300, SCP/DCP VD, OR flow) do not differ between the two T2DM subgroups.

Table S7. Outlier screening and sensitivity analysis.

Outlier threshold	N (total)	N (T2DM)	N (Control)	XGBoost CV AUC
< 1 flagged parameters	159	109	50	0.918
< 2 flagged parameters	203	140	63	0.924
< 3 flagged parameters	217	152	65	0.926
**< 5 flagged parameters (primary)**	**221**	**155**	**66**	**0.927**
No outlier removal	226	160	66	0.921

Sensitivity of primary XGBoost AUC to outlier-screening threshold. The 3×IQR rule with threshold of 5 flagged parameters (primary analysis, bolded row) gives the best balance of cohort preservation and noise reduction. All thresholds yield AUC ≥ 0.918, confirming the robustness of the result.

Table S8. Pre-specified logistic-regression panels (A–H) performance.

Panel	Features	10-fold CV AUC
A: FD-300 alone	FD-300	0.786 ± 0.144
B: SCP parafoveal VD	SCP VD – Parafovea	0.791 ± 0.121
C: DCP parafoveal VD	DCP VD – Parafovea	0.819 ± 0.096
D: Conventional VD	SCP + DCP whole image	0.848 ± 0.076
E: D + FAZ circularity	D + FAZ circularity index	0.851 ± 0.079
F: D + CC flow area	D + choriocapillaris flow area	0.863 ± 0.072
G: D + OR flow area	D + outer retina flow area	0.859 ± 0.074
**H: Integrative 5-feature**	FD-300 + DCP parafovea + FAZ circ + CC + OR	**0.873 ± 0.084**

Pre-specified panels evaluated with logistic regression and 10-fold stratified cross-validation. Panel H, the integrative clinically interpretable panel, achieves the best AUC in this family.

Table S9. Full SHAP top-20 ranking for the XGBoost classifier.

Rank	OCTA feature	Mean |SHAP|	XGBoost gain
**1**	**FD-300 (foveal density %)**	**0.813**	0.0270
**2**	**CC – Flow ratio**	**0.722**	0.0114
**3**	**DCP VD – Parafovea I-hemi**	**0.659**	0.0337
**4**	**DCP VD – Parafovea**	**0.651**	0.1353
**5**	**Retinal thickness – Fovea**	**0.580**	0.0112
6	Retinal thickness – Parafovea Superior	0.579	0.0277
7	DCP Thickness – Parafovea Superior	0.390	0.0166
8	DCP VD – Parafovea Nasal	0.354	0.0286
9	DCP VD – Whole image	0.339	0.0196
10	Retinal thickness – Parafovea Nasal	0.321	0.0062
11	OR – Flow ratio	0.277	0.0122
12	DCP VD – Parafovea Temporal	0.257	0.0285

Rank	OCTA feature	Mean |SHAP|	XGBoost gain
13	SCP VD – Parafovea Temporal	0.214	0.0313
14	SCP VD – Parafovea Superior	0.202	0.0044
15	Retinal thickness – Perifovea S-hemi	0.198	0.0056
16	FAZ perimeter (mm)	0.198	0.0053
17	DCP Thickness – Parafovea	0.144	0.0728
18	SCP Thickness – Fovea	0.135	0.0080
19	SCP Thickness – Parafovea Superior	0.130	0.0116
20	DCP Thickness – Parafovea I-hemi	0.108	0.0862

Full SHAP top-20 ranking. Top-5 features in bold. XGBoost gain = fraction of total tree-split gain attributable to the feature during training.

Supplementary Figures

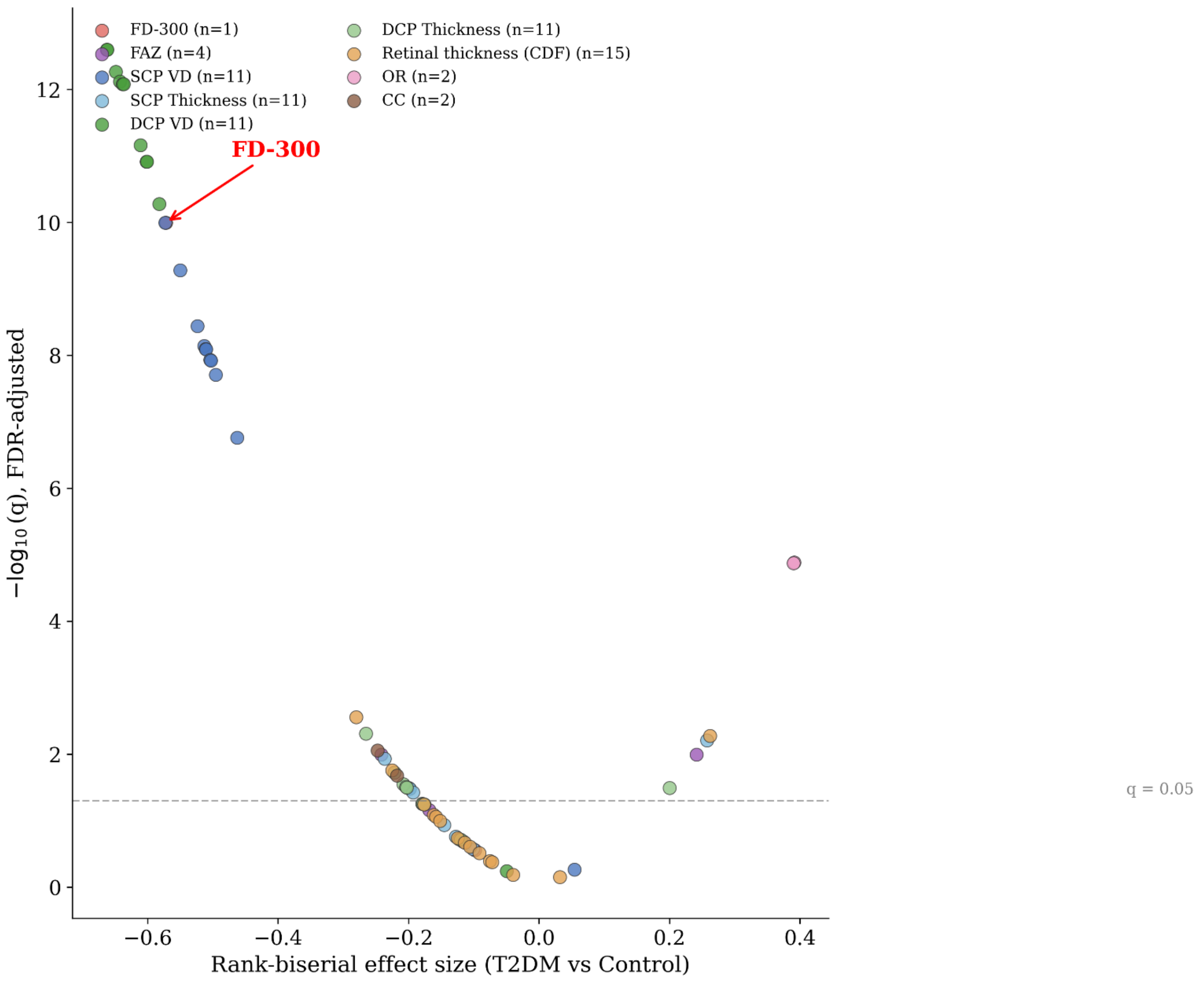

Figure S1. Volcano plot of all 68 OCTA parameters: rank-biserial effect size (T2DM versus Control) on the horizontal axis versus $-\log_{10}$(FDR-adjusted q-value) on the vertical axis, colour-coded by biomarker family. Each dot represents one OCTA parameter. The horizontal dashed line marks the $q = 0.05$ significance threshold. Colour encoding: red = FD-300 ($n = 1$); purple = FAZ family ($n = 4$); dark blue = SCP VD ($n = 11$); light blue = SCP Thickness ($n = 11$); dark green = DCP VD ($n = 11$); light green = DCP Thickness ($n = 11$); orange = retinal-thickness map ($n = 15$); pink = outer retina ($n = 2$); brown = choriocapillaris ($n = 2$). The DCP vessel-density family clusters at the strongest effect-size region ($r\_b \approx -0.65$, $q \approx 10^{-12}$, top left). FD-300 is highlighted with a red annotated arrow. Outer-retina flow parameters are the only significant positive-r_b findings, sitting in isolation on the right-hand side.

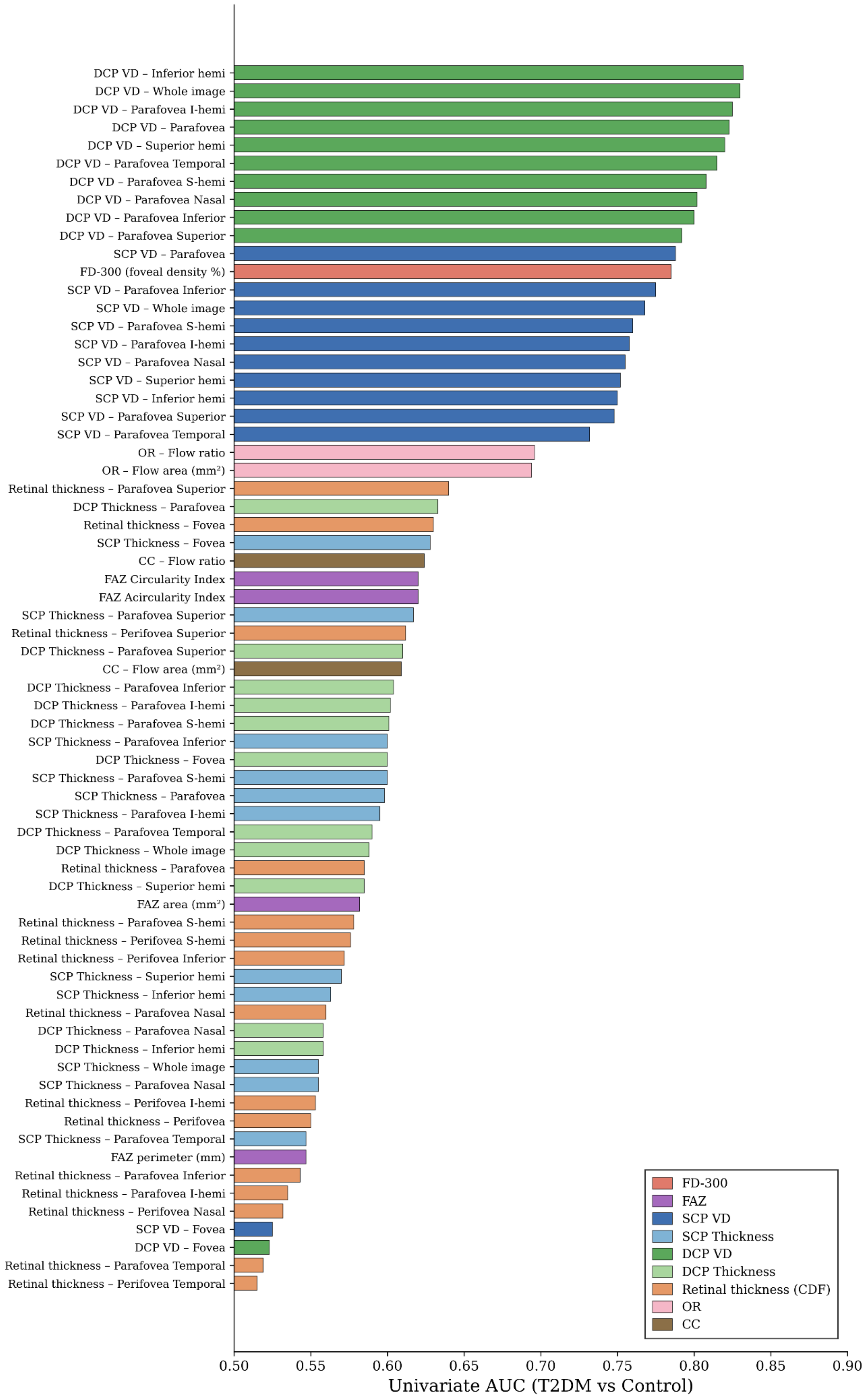

DCP VD – Inferior hemi
DCP VD – Whole image
DCP VD – Parafovea I-hemi
DCP VD – Parafovea
DCP VD – Superior hemi
DCP VD – Parafovea Temporal
DCP VD – Parafovea S-hemi
DCP VD – Parafovea Nasal
DCP VD – Parafovea Inferior
DCP VD – Parafovea Superior
SCP VD – Parafovea
FD-300 (foveal density %)
SCP VD – Parafovea Inferior
SCP VD – Whole image
SCP VD – Parafovea S-hemi
SCP VD – Parafovea I-hemi
SCP VD – Parafovea Nasal
SCP VD – Superior hemi
SCP VD – Inferior hemi
SCP VD – Parafovea Superior
SCP VD – Parafovea Temporal
OR – Flow ratio
OR – Flow area (mm²)
Retinal thickness – Parafovea Superior
DCP Thickness – Parafovea
Retinal thickness – Fovea
SCP Thickness – Fovea
CC – Flow ratio
FAZ Circularity Index
FAZ Acircularity Index
SCP Thickness – Parafovea Superior
Retinal thickness – Perifovea Superior
DCP Thickness – Parafovea Superior
CC – Flow area (mm²)
DCP Thickness – Parafovea Inferior
DCP Thickness – Parafovea I-hemi
DCP Thickness – Parafovea S-hemi
SCP Thickness – Parafovea Inferior
DCP Thickness – Fovea
SCP Thickness – Parafovea S-hemi
SCP Thickness – Parafovea
SCP Thickness – Parafovea I-hemi
DCP Thickness – Parafovea Temporal
DCP Thickness – Whole image
Retinal thickness – Parafovea
DCP Thickness – Superior hemi
FAZ area (mm²)
Retinal thickness – Parafovea S-hemi
Retinal thickness – Perifovea S-hemi
Retinal thickness – Perifovea Inferior
SCP Thickness – Superior hemi
SCP Thickness – Inferior hemi
Retinal thickness – Parafovea Nasal
DCP Thickness – Parafovea Nasal
DCP Thickness – Inferior hemi
SCP Thickness – Whole image
SCP Thickness – Parafovea Nasal
Retinal thickness – Perifovea I-hemi
Retinal thickness – Perifovea
SCP Thickness – Parafovea Temporal
FAZ perimeter (mm)
Retinal thickness – Parafovea Inferior
Retinal thickness – Parafovea I-hemi
Retinal thickness – Perifovea Nasal
SCP VD – Fovea
DCP VD – Fovea
Retinal thickness – Parafovea Temporal
Retinal thickness – Perifovea Temporal
FD-300
FAZ
SCP VD
SCP Thickness
DCP VD
DCP Thickness
Retinal thickness (CDF)
OR
CC
0.50
0.55
0.60
0.65
0.70
0.75
0.80
0.85
0.90
Univariate AUC (T2DM vs Control)

Figure S2. Univariate ROC-AUC bar chart for all 68 OCTA parameters, ranked by descending discriminative power for the T2DM-versus-Control task. Each horizontal bar represents one parameter; bar colour encodes biomarker family using the same palette as Figure S1 (legend, bottom right). Bars are ordered along the vertical axis from highest AUC at the top to lowest at the bottom. The horizontal axis starts at AUC = 0.50, the no-discrimination reference. The top ten positions are dominated by DCP vessel-density parameters (AUC ≈ 0.79–0.83), followed by SCP parafoveal vessel density and FD-300 (AUC ≈ 0.74–0.79). Outer-retina flow parameters represent the strongest non-vascular discriminators (AUC ≈ 0.69). Retinal-thickness, FAZ-geometry, SCP/DCP thickness, and choriocapillaris parameters fall in the moderate-to-low range (AUC ≈ 0.52–0.64).

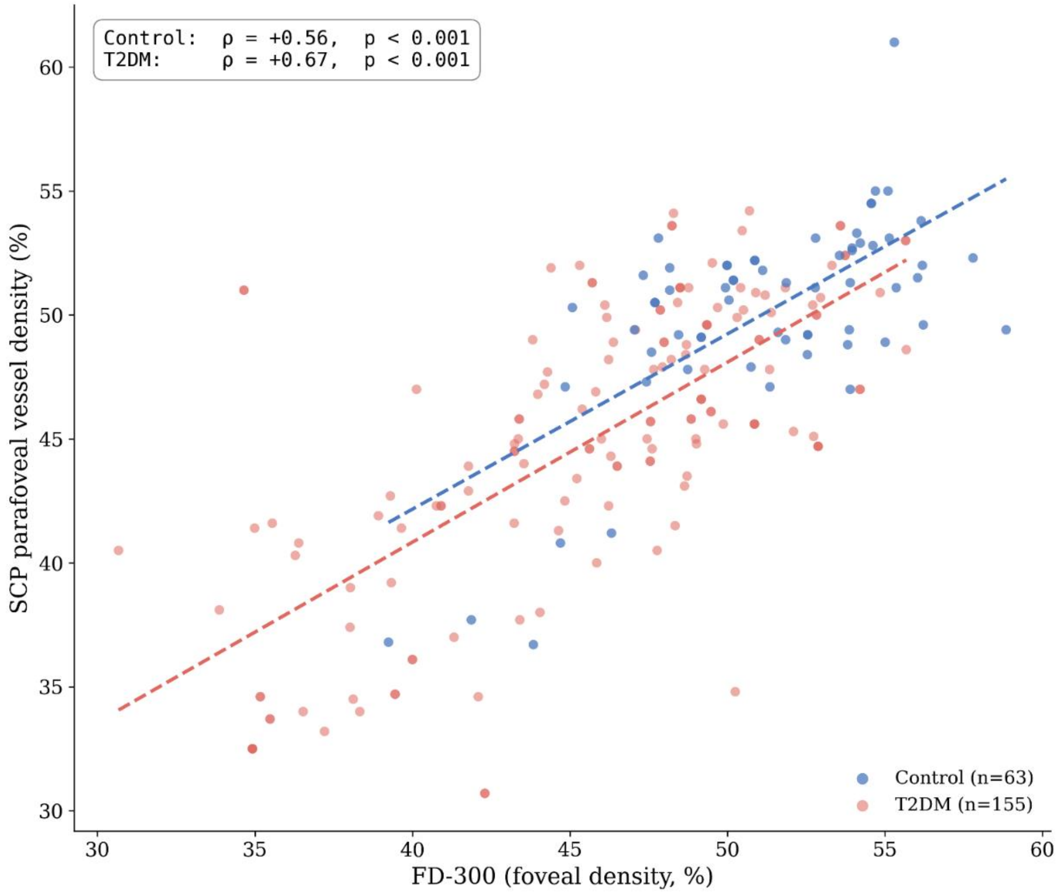

Figure S3. Relationship between FD-300 and SCP parafoveal vessel density, stratified by diagnostic group. Scatter plot of the two most discriminative perifoveal/parafoveal biomarkers. Each dot represents one eye, with Control eyes shown in blue (n = 66) and T2DM eyes in red (n = 155). Per-group linear regression lines are shown as dashed lines in the corresponding colour. Spearman rank-correlation coefficients are displayed in the top-left annotation box (Control ρ = +0.56, $p < 0.001$; T2DM ρ = +0.67, $p < 0.001$.

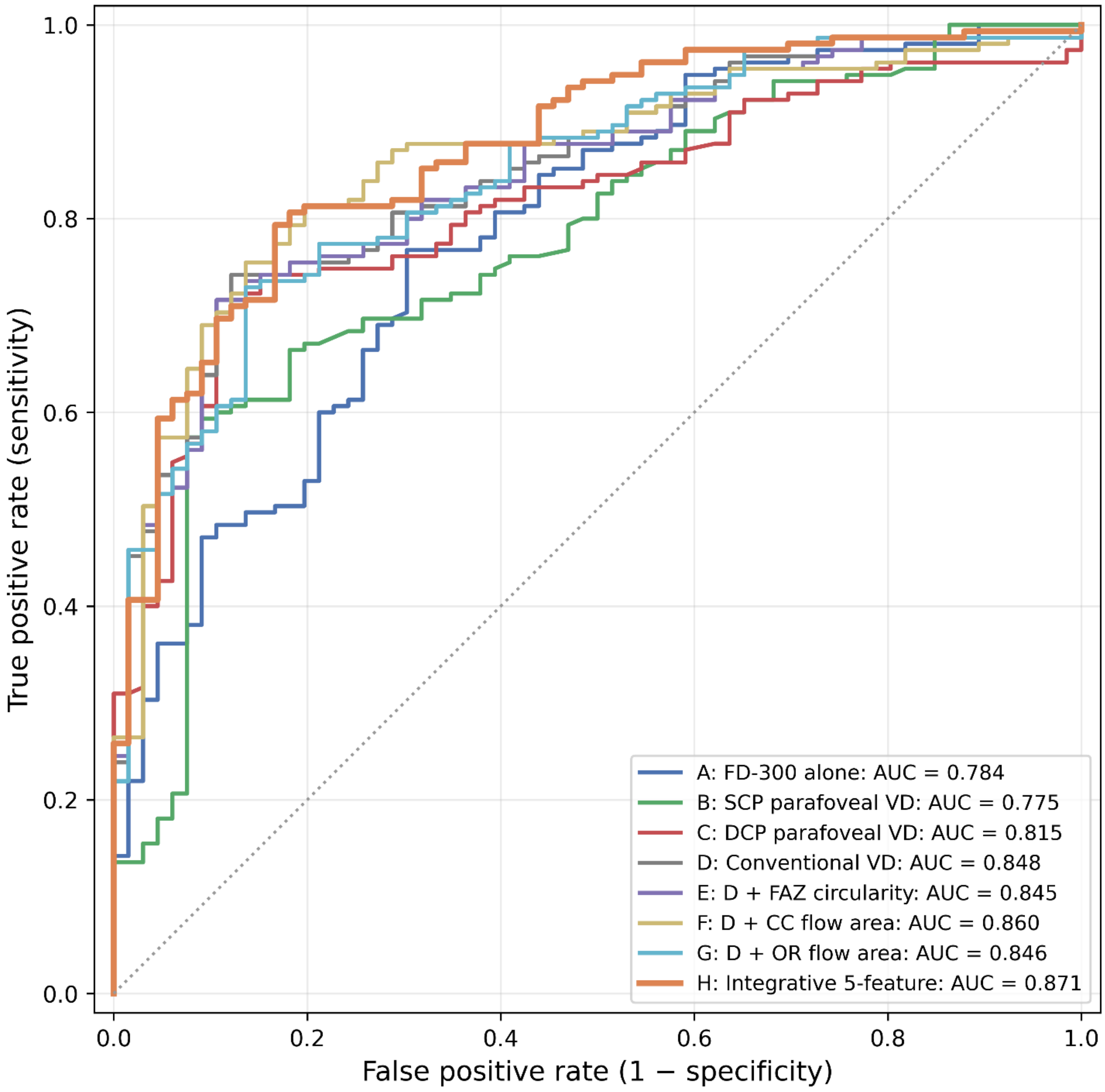

Figure S4. Figure S4. Receiver-operating-characteristic (ROC) curves for the eight pre-specified logistic-regression panels (A–H) listed in Table S8, under patient-grouped cross-validation. All curves derive from the pooled out-of-fold predictions of patient-grouped 10-fold cross-validation on the 221-eye analytic cohort, using the same random seed as the main-text machine-learning analysis. Panel labels and pooled out-of-fold AUC values are shown in the legend. The integrative five-feature panel H (orange, AUC = 0.871) achieves the highest AUC in this family, above the conventional two-feature panel D (grey, AUC = 0.848); the single-feature panels A–C have the lowest AUC values. The diagonal dotted line represents a non-informative classifier.

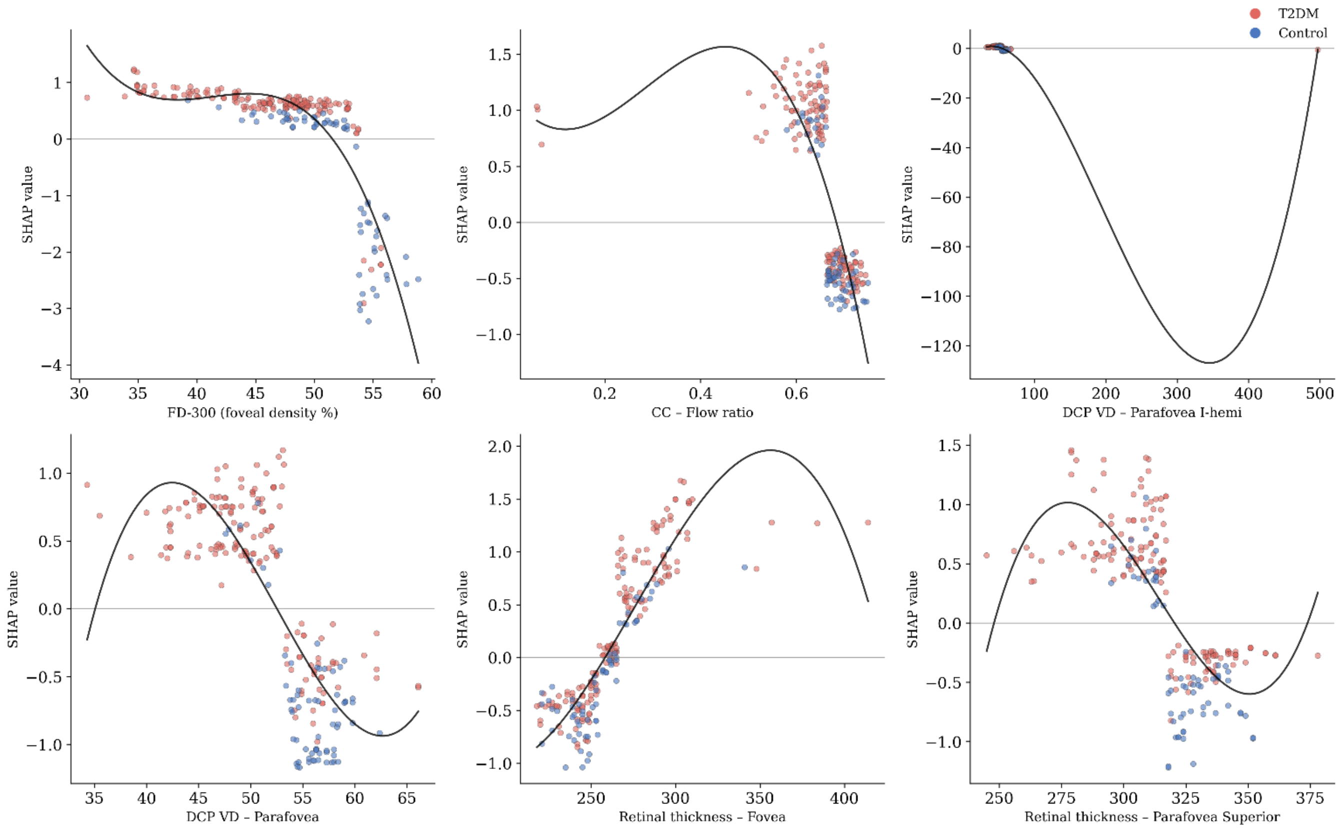

Figure S5. SHAP dependence plots for the top six features identified in the XGBoost classifier. Each of the six panels plots, for one feature, the per-eye SHAP value (y-axis) against the raw feature value (x-axis). Dots are coloured by diagnostic group (red = T2DM, n = 155; blue = Control, n = 66), and a smoothed cubic-polynomial fit is overlaid as a solid black line. A horizontal grey line at SHAP = 0 marks the neutral decision boundary. Features shown, in the order of the SHAP ranking, are: (1) FD-300, (2) CC flow ratio, (3) DCP VD parafoveal I-hemi, (4) DCP VD parafoveal, (5) retinal thickness fovea, (6) retinal thickness parafoveal superior. Each dependence curve exhibits a broadly monotonic relationship with a pronounced transition around the diagnostically informative threshold–FD-300 near 50%, CC flow ratio near 0.67, DCP parafoveal VD near 54%.
